# *Atriplex portulacoides* L*.* derived phytochemicals mitigate acetic acid induced colitis in rats via orchestrating Nrf2/Keap1 signalling and LncRNAs gene expression

**DOI:** 10.1007/s10787-026-02332-0

**Published:** 2026-07-30

**Authors:** Maha Hassan, Marwa M. Mona, Marwa M. Abd-Elsalam, Mona El-Aasr, Amal Kabbash, Ghada Attia

**Affiliations:** 1 Department of Pharmacognosy, Faculty of Pharmacy, Tanta University, Tanta, 31527 Egypt; 2 Medical Biochemistry and Molecular Biology Department, Faculty of Medicine, Kafrelsheikh University, Kafrelsheikh, 33511 Egypt; 3 Histology and Cell Biology, Faculty of Medicine, Kafrelsheikh University, Kafrelsheikh, 33511 Egypt

**Keywords:** *Atriplex portulacoides* L., LC–ESI–MS/MS, Ulcerative colitis, Nrf2/Keap1 signalling pathway, TNF-*α*, NF-Κb, LncRNAs

## Abstract

**Background:**

This study is the first to elucidate the mechanistic actions of *Atriplex portulacoides* L. methanolic extract (APME) as a natural treatment of acetic acid-induced ulcerative colitis on rat model.

**Methods:**

Phytochemical metabolic profiling of APME was determined through the determination of total phenolic and flavonoid contents, alongside negative mode LC–ESI–MS/MS analysis. Furthermore, chromatographic and spectroscopic investigations were employed to fractionate and structurally characterize the metabolites present within APME fractions. The therapeutic potential of APME in UC was evaluated by integrating histological and immunohistochemical analyses. Quantification of oxidative stress, inflammation-related, and lncRNAs (FENDRR and Neat1) gene expression was established.

**Results:**

APME was evaluated for its total phenolic content and total flavonoid content. LC–ESI–MS/MS analysis in negative ionization mode identified 35 bioactive metabolites within APME. Stigmasterol (1) and 20-Hydroxyecdysone (2) were isolated by column chromatography from the *n*-hexane and ethyl acetate fractions, respectively, and characterized using spectroscopic analysis. APME dose-dependently and significantly (*p* < 0.05) reduced the pro-inflammatory cytokine TNF-α, mitigated malondialdehyde levels, and restored total antioxidant capacity in colonic tissue compared to the untreated ulcerative group. APME`s treatments markedly upregulated the mRNA expression levels of Nrf2 and HO-1, while concurrently suppressing NF-κB mRNA expression. Also, it showed a regulatory effect on FENDRR and Neat1. Histological, immunohistochemical, and morphometric assessments further supported these findings, demonstrating substantial improvements in colonic architecture and cellular integrity.

**Discussion:**

Notably, APME represents a potential antioxidant and anti-inflammatory therapeutic candidate for UC. It modulates FENDRR and Neat1, revealing promising targets for IBD therapy.

**Supplementary Information:**

The online version contains supplementary material available at https://doi.org/10.1007/s10787-026-02332-0.

## Introduction

Immune-mediated inflammatory diseases are diverse disorders marked by abnormal immune responses and dysregulated immunological pathways, affecting various body organs. Gastrointestinal IMIDs overlap, complicating diagnosis, including inflammatory bowel disease [IBD], coeliac, vasculitis, gastroenteritis, sarcoidosis, colitis, and microscopic colitis. IBD primarily consists of two distinct clinical entities: Crohn’s disease [CD] and ulcerative colitis [UC] (Vuyyuru et al. 2022).

The pathogenesis of IBD is complex and not yet fully understood. It involves multifactorial interplay among genetic susceptibility, environmental factors, immune system dysregulation, and disturbances in the composition and function of the gut microbiota (Zhang and Li 2014).

The core mechanism underlying IBD pathogenesis involves an abnormal immune response to the intestinal microbiota. In healthy individuals, the immune system maintains homeostasis by distinguishing between harmless commensal organisms and harmful pathogens. However, in those with genetic susceptibility, this regulatory balance is disrupted. Environmental factors—such as diet, infections, and stress—can intensify this imbalance, promoting an exaggerated inflammatory response within the gut (Abdulla and Mohammed 2022).

NF-κB is a key inducible transcription factor with critical roles in numerous physiological and pathological processes, making it a prominent target in therapeutic strategies, particularly in cancer and inflammatory diseases (Li et al. 2015; Oeckinghaus et al. 2011). Although NF-κB exhibits complex and multifaceted functions, it does not operate in isolation. Instead, it engages in extensive crosstalk with other signaling cascades to mediate a wide range of cellular responses (Attiq et al. 2018). Its activation can be triggered by diverse stimuli, including pro-inflammatory cytokines, oxidative stress, and microbial infections, leading to downstream effects on cell proliferation, differentiation, survival, immune modulation, and intercellular communication (Gerondakis et al. 1999; Mattson and Meffert 2006).

Experimental models, particularly in mice, have demonstrated the importance of Rel/NF-κB and IκB family members in regulating these pathways through genetic manipulation such as transgenic and knockout approaches (Courtois and Gilmore 2006). Disruption of NF-κB signaling has been implicated in the pathogenesis of a variety of disorders, including autoimmune and inflammatory diseases, metabolic syndromes, and cancers (Taniguchi and Karin 2018). Oxidative stress is a central feature of IBD pathophysiology, where reactive oxygen species [ROS] induce lipid peroxidation, protein oxidation, and DNA damage in intestinal epithelial cells. Several transcription factors, including nuclear factor erythroid 2-related factor 2 [*Nrf2*], heme oxygenase-1 [*HO-1*], and *NF-κB*, regulate redox homdamage in the immune system and inflammatory signaling. While *Nrf2* and *HO-1* exert protective roles by mitigating oxidative stress, *Kelch-like ECH-associated protein 1* [*Keap1*] inhibits *Nrf2* activity, exacerbating cellular injury (Li et al. 2015). Additionally, *β-actin* serves as a housekeeping gene in gene expression studies related to these pathways.

Approximately 30% of patients exhibit primary non-responsiveness to anti-TNFα therapy. Moreover, among initial responders, up to 10% experience a loss of therapeutic efficacy annually. Existing pharmacological options are also associated with considerable risks, including infections and malignancies. These limitations highlight a critical unmet need for the development and clinical integration of more effective therapeutic agents or combination regimens with improved safety profiles (Hazel and O'Connor 2020).

Plant-derived natural products have long been recognized for their beneficial effects on human health, historically utilized for purposes such as pain relief, wound healing, gastrointestinal support, and various other therapeutic applications (Davila and Papada 2023). In recent decades, these natural compounds have garnered significant scientific attention, prompting extensive research focused on the identification, isolation, quantification, and evaluation of their bioactive constituents and associated biomedical properties.

*Atriplex portulacoides* L. (AP) is a halophytic perennial shrub commonly known as sea purslane. It is widely distributed across African, European, and North American coasts, with limited presence along some Asian shores (Kubiak-Martens et al. 2015). It belongs to the family Amaranthaceae and is one of the eighteen species of the genus *Atriplex* found in Egypt. In vivo investigation has reported the therapeutic potential of some *Atriplex* species in the management of ulcerative colitis (Abd El Raheim et al. 2013).

AP is traditionally used as animal fodder and as a source of nutrition (Zanella and Vianello 2020). Hamada (2025) revealed the anti-cytogenotoxic effect of AP`s crude extract in mice bone marrow (Hamada et al. 2025).

Recent studies highlight long non-coding RNAs [lncRNAs] in IBD pathogenesis, particularly FENDRR [FOXF1 adjacent non-coding developmental regulatory RNA] and Neat1 [nuclear paraspeckle assembly transcript 1]. Neat1 drives cytokine expression, mitochondrial stress, and apoptosis, while FENDRR regulates macrophage activation, suggesting a biomarker for gastrointestinal tumorigenesis (He et al. 2019).

Herein, a phytochemical metabolic profiling of APME was accomplished via total phenolic, total flavonoid, and liquid chromatography- electrospray negative mode ionization tandem mass spectrometric [LC–ESI–MS/MS] analyses. Additionally, chromatographic and spectroscopic studies were conducted to separate and identify metabolites present in APME’s fractions. We evaluated the unrevealed therapeutic potential of APME in the treatment of UC via integrating histological and immunohistochemical assessments with quantifying the oxidative stress and inflammation-related gene expression. We also explored for the first time the modulation of FENDRR and Neat1 expressions by APME as potential molecular targets in IBD therapy.

## Materials and methods

### Plant material for APME chromatographic and biological studies

Aerial parts of *A. portulacoides* L. were collected from Kafr El-Sheikh Baltim coastal road, Egypt, in January 2019. The plant was kindly verified by Prof. Dr. Ahmed Sharaf eldin, Professor of Plant Ecology, Botany Department, Faculty of Science, Tanta University. Voucher sample No. PGAAP012019 was deposited at the herbarium of the Pharmacognosy department, Faculty of Pharmacy, Tanta University. The plant material was shade-dried, then ground into powder and kept in firmly closed containers.

2.8 kg of dried AP aerial parts powder were extracted through maceration (5 × 16 L) using methanol until complete exhaustion. The extract was then concentrated under reduced pressure using a rotary evaporator, yielding 120 g of APME for phytochemical and biological studies.

A total of 20 g of APME was suspended in 0.5% methyl cellulose to prepare dosing solutions corresponding to two final concentrations: 100 mg/kg and 200 mg/kg of rat body weight for use in the biological experiments.

Instruments and reagents for APME chromatographic and biological studies (Supplementary materials).

Total phenolics & total flavonoids examination of APME: (Supplementary materials).

LC–ESI–MS /MS Analysis of APME: (Supplementary materials).

Chromatographic separation and spectroscopic identification of metabolites from APME’s fractions: (Supplementary materials).

### Materials for the APME biological study

Induction of Ulcerative Colitis: (Supplementary materials).

#### Experimental animals

The study was conducted using 50 healthy adult male albino rats, each weighing between 200 and 250 g. All animals were housed under controlled environmental conditions (12-h light–dark cycle; 24 ± 2 °C; 60–70% relative humidity) and acclimatized for two weeks before the initiation of the experiment.

#### Ethics statement

Animal handling was strictly adhered to the approved guidelines of the research ethics committee for the care and use of laboratory animals, at the Faculty of Pharmacy, Kafrelsheikh University (approval number of KFS-IACUC/301/2025). Following the ethical standards, the Egyptian guide for the care and use of laboratory animals has been used. The local ethics committee allowed the experimental protocol, which was fully committed to the ARRIVE criteria for reporting in vivo investigations and employing laboratory animals. The animals were humanely euthanised by administration of an intraperitoneal injection of sodium pentobarbital (50 mg/kg) at the end of the experiment.

#### Experimental design

The animals were randomly allocated into five groups, with 10 rats in each:

- **Group 1** (Control Group): This group was further subdivided into two subgroups (n = 5 each):
- Subgroup I received the vehicle control (methyl cellulose) orally at a volume of 1 mL daily for 10 consecutive days. Also, the control group was given a single intra-rectal (IR) dose of 2 mL of 0.9% normal saline on the induction day.
- **Group 2** (APME 200 mg/kg): Rats were treated orally with 200 mg/kg of APME dissolved in 0.5% methyl cellulose for three days prior to colitis induction and continued for six additional days, including the day of induction. On the induction day, they also received a single intra-rectal instillation of 2 mL of 0.9% saline.
- **Group 3** (Acetic Acid Group): Ulcerative colitis was induced in this group by intra-rectal administration of 2 mL of 3% acetic acid in normal saline. No treatment was administered to this group.
- **Group 4** (APME 100 mg/kg + Acetic Acid): Rats received 100 mg/kg of APME orally for three days prior to acetic acid induction and continued for four days post-induction.
- **Group 5** (APME 200 mg/kg + Acetic Acid): This group received the higher dose of APME (200 mg/kg) following the same regimen as Group 4.

On the 10th day of the experiment, all animals were humanely euthanised under deep anaesthesia induced by an intraperitoneal injection of sodium pentobarbital (50 mg/kg). Colonic tissues were then carefully excised, rinsed with saline, and processed for subsequent histological and immunohistochemical evaluations, following established protocols for experimental colitis models (Owusu et al. 2020).

#### Histological examination

For histopathological evaluation, colonic tissues from all experimental groups were fixed in 10% neutral buffered formalin, dehydrated, and embedded in paraffin to prepare tissue blocks. Sections were cut at a thickness of 5 µm and processed for histological analysis. The sections were stained with hematoxylin and eosin (H&E) to assess general tissue morphology, inflammatory infiltration, epithelial damage, and structural integrity of the colonic mucosa, following standard histological procedures (Bancroft and Layton 2010; Millar et al. 1996).

Immunohistochemical Study: (Supplementary materials).

Morphometric Analysis: (Supplementary materials).

Quantitative Real-Time PCR (qRT-PCR): (Supplementary materials).

RNA Extraction: (Supplementary materials).

Primer Design and PCR Reaction: (Supplementary materials).

Gene Expression Analysis: (Supplementary materials).

Measurement of TNF-α: (Supplementary materials).

Assessment of Oxidative Stress Biomarkers in Colon Homogenates: (Supplementary materials).

Assessment of Gene Expression Levels of β–Actin, Nrf2, Keap1, NF-κB, and HO-1 mRNA: (Supplementary materials).

Assessment of Gene Expression Levels of Fendrr and Neat1 lncRNAs: (Supplementary materials).

Statistical analysis: (Supplementary materials).

## Result

### Phytochemical investigation of APME

#### Total phenolic and flavonoid contents

APME was found to contain 10.89 ± 0.95 mg GAE/g extract and 12.8 ± 0.84 mg RE/g extract of total phenolic and flavonoid compounds, respectively.

#### APME LC–ESI–MS/MS-based metabolic profiling

Tentative metabolic profiling of APME using negative mode LC–ESI–MS/MS identified 35 metabolites of several phytochemical classes, including organic acids, cinnamic acid derivatives, flavonoid aglycones and glycosides, biflavonoids, carbohydrates, purines, pyrimidines, amino acids, and volatile compounds (Table S1 and Figure S1). These results were in coherence with Liberian data and previously reported literatures.

##### Organic acids

Five deprotonated molecular ion peaks appeared at *m/z* 133.01425 (Abdl Aziz et al. 2024), 175.0612 (Sri Harsha et al. 2018), 165.05573 (Abdl Aziz et al. 2024), 137.02441, and 153.01933 (Collins 2022), referring to D-(+)-malic acid **1**, 2-isopropylmalic acid **2**, D-3-Phenyllactic acid **3, p**-hydroxybenzoic acid **4**, 2,5-dihydroxybenzoic acid **5**, respectively.

##### Cinnamic acid derivatives

Two characteristic phenolic acids were recognized as caffeic acid **6** and rosmarinic acid **7** with [M-H] ¯ peak at *m/z* 179.03499 (Abdl Aziz et al. 2024) and 359.07724 (Stanojević et al. 2018), respectively.

##### Flavonoids and their glycosides

Two flavonol-type aglycones were detected as myricetin **8** at *m/z* 317.0303 and 3,5, 7-trihydroxy-4’-methoxyflavone **15** at *m/z* 299.05612 (Abdl Aziz et al. 2024). However, four flavonol -*O*-glycosides of variant glycone moieties were detected as kaempferol-3-*O*-*α*-L-rhamnoside **10**, isorhamnetin-3-*O*-rutinoside **11**, kaempferol-7-O-neohesperidoside **13**, kaempferol-3-Glucuronide **14**. These deprotonated molecular ion peaks appeared at *m/z* 431.09836 (Abdl Aziz et al. 2024), 623.16174 (Abdl Aziz et al. 2024), 593.15118, and 461.07254 (Fabre et al.^2001^; Yuan et al. 2008), respectively. Moreover, the flavone`s glycoside baicalein-7-*O*-glucuronide **9** was detected at *m/z* 445.07764 (Abdl Aziz et al. 2024), while the hydroxychalcone glycoside okanin-4’-*O*-glucoside **12** appeared at *m/z* 449.10895. The only detected biflavonoid at *m/z* 577.13513 was procyanidin B2 **16**.

##### Carbohydrates and sugar derivatives

Different carbohydrates were observed as the three sugar acids L-( +)-Tartrate **17**, gluconate **18**, and mucate **19**, the sugar alcohol galactinol dihydrate **20**, the disaccharide sucrose **21**, and the oligosaccharides nystose trihydrate **22**. Their deprotonated peaks were measured at *m/z* 149.00916, 195.05103 (Abdel Aziz et al. 2024), 209.0309 (El-Zawahry et al. 2025), 378.10895 (Abdelgawad et al. 2024), 341.10895 (Taylora et al. 2005), and 665.2783, respectively.

##### Purines and pyrimidines

The purine nucleosides inosine **23** and xanthosine **24** appeared at *m/z* 267.07349 and 283.06842 (Li et al. 2009), respectively. Other pyrimidine nucleosides were identified as, 2’-Deoxyuridine-5’-triphosphate sodium salt **25**, uridine 5'-monophosphate **26**, UDP-xylose **27**, 2’-deoxyuridine-5’-triphosphate sodium salt uridine 5'-diphosphate **28**, uridine 5'-diphosphate **29**, thymidine-3’,5’ cyclic monophosphate sodium salt **30** and uridine 5’-diphosphoglucuronic acid **31**, These pyrimidine derivatives showed [M-H] ¯ peaks values at *m/z* 307.0423, 323.1175, 535.03717, 466.966, 303.03876 and 579.02704, respectively.

##### Miscellaneous metabolites

The volatile compound *ˠ*-Terpinene **32** was identified at *m/z* 135.11792 (Ryhage and Sydow 1963), while the two amino acids L-5- oxoproline **33**, trans-4-hydroxy-L-proline **34**, and L-*β*-homoisoleucine **35** were detected at *m/z* 128.03532, 130.05096, and 144.04691, respectively.

### Isolation and spectroscopic identification of components at AP fractions (supplementary material)

**Fig. 1:**
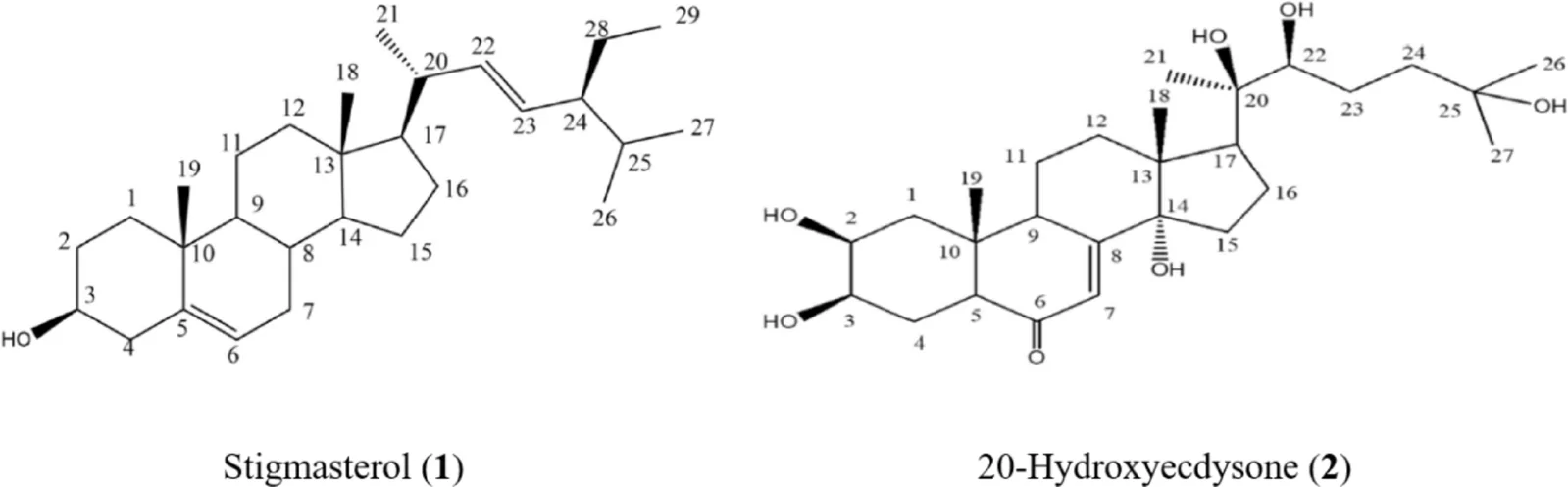
Compounds isolated from AP fractions

APME fractionation, chromatography-based purification, and spectral analysis (supplementary material) revealed the isolation of stigmasterol **1** (20 mg of an amorphous white powder) and 20-hydroxyecdysone **2** (8 mg of a yellowish amorphous solid) from its n-hexane and ethyl acetate fractions, respectively (Fig. 1).

### In Vivo evaluation of APME efficacy in UC Treatment

Guided by the high LD50 of an anti-ulcer activity of some species in the genus *Atriplex* (Abd El Raheim et al. 2013), no animal mortality was observed, indicating that APME was well-tolerated at the tested doses. The control group showed no significant differences in histopathological findings or biochemical outcomes. Therefore, they were collectively designated as the unified control group for subsequent analyses.

Similarly, animals in the APME-only treatment group (Group 2), which did not undergo colitis induction, showed no noticeable alterations in the histological architecture of the colon. Additionally, no significant differences were found between this group and the control group regarding all measured parameters. These results confirm that APME, when administered alone, exerted no adverse structural or inflammatory effects on colonic tissues, supporting its safety in vivo.

### Light microscopic assessment of colonic tissue (H&E staining)

**Fig. 2:**
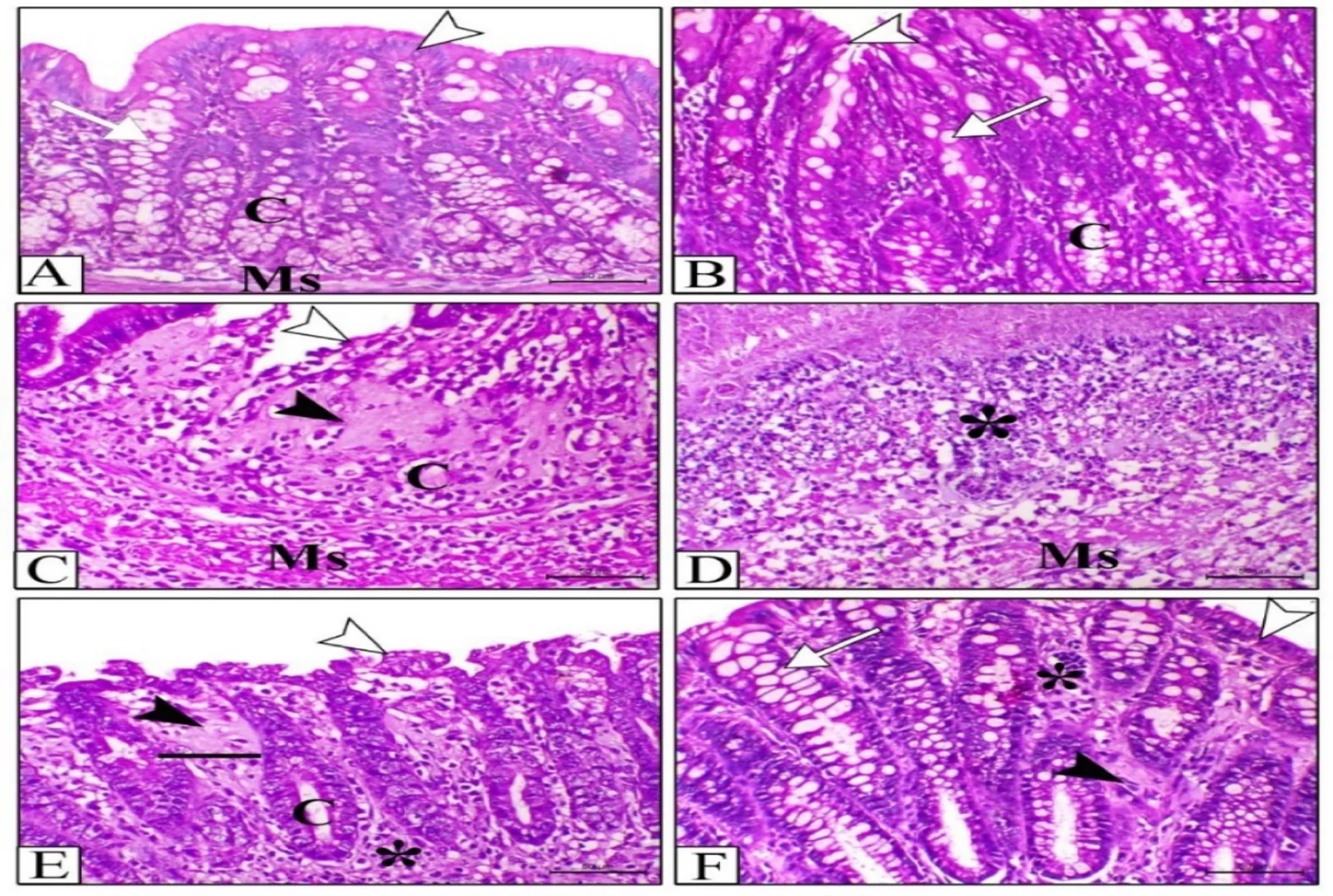
Hematoxylin and eosin (H&E)-stained sections of colonic mucosa from different experimental groups; A-F (magnification × 200, scale bar = 50 μm). **A** Group 1 (control) and **B** Group 2 (200 mg/kg APME) show normal mucosal architecture with numerous, regularly arranged, and closely packed crypts of Lieberkühn **C** extending through the mucosa and resting on the muscularis mucosa (Ms). The surface epithelium and crypt lining consist mainly of columnar epithelial cells (white arrowheads) with apical brush borders and basal nuclei, along with abundant goblet cells (arrows) displaying vacuolated cytoplasm and basal nuclei. Goblet cells increase in number toward the crypt base, while columnar cells become fewer. The lamina propria contains resident immune cells, mainly lymphocytes. **C**, **D** Group 3 (colitis-induced) exhibits complete crypt destruction (**C**), loss of surface epithelium (white arrowhead), absence of goblet cells, homogenous acidophilic areas in the lamina propria (black arrowhead), and extensive infiltration of inflammatory cells (star) reaching the muscularis mucosa. **E** Group 4 (100 mg/kg APME + colitis) shows focal crypt distortion, a marked reduction in goblet cells (white arrowheads), widened intercryptal spaces (line), homogenous acidophilic areas (black arrowhead), and mononuclear cell infiltration in the lamina propria (star). **F** Group 5 (200 mg/kg APME + colitis) reveals preserved mucosal architecture with intact crypts, continuous surface epithelium (white arrowhead), numerous goblet cells (arrows), minimal acidophilic change (black arrowhead), and mild mononuclear infiltration (star)

Histological evaluation of hematoxylin and eosin (H&E)-stained sections from the control group (G1) revealed intact colonic architecture characterized by well-organized, tightly packed simple tubular glands (crypts of Lieberkühn) extending into the muscularis mucosa. The crypts were lined by columnar absorptive epithelial cells displaying apical brush borders, basal oval nuclei, and numerous goblet cells with vacuolated cytoplasm. Occasional intraepithelial lymphocytes were also observed, reflecting normal mucosal immune surveillance (Fig. 2A. Similarly, the colonic sections of the APME-treated rats (G2) display preserved histological features with no evident alterations in crypt architecture or goblet cell distribution (Fig. 2B, supporting the non-toxic profile of APME.

In contrast, H&E-stained sections from the ulcerative colitis group (G3) demonstrated extensive mucosal damage. The colonic crypts were severely disrupted, with a complete loss of surface epithelium, marked depletion or total absence of goblet cells, and areas of mucosal detachment and hemorrhage (Fig. 2C. Dense mononuclear cellular infiltrates were observed within the lamina propria, extending into the muscularis mucosa, reflecting active inflammation (Fig. 2D).

Colonic sections from the G4 group (100 mg/kg APME + A.A.) exhibited partial protection, with no frank ulceration but evidence of focal crypt architectural distortion and intercryptic space widening. A limited number of goblet cells were identified, and moderate mononuclear infiltration extended into the muscularis mucosa (Fig. 2E, indicating an intermediate anti-inflammatory response.

Notably, treatment with the higher dose of APME (G5; 200 mg/kg + A.A.) resulted in near-complete preservation of colonic architecture. Crypt integrity was restored, surface epithelium remained continuous, and goblet cell populations were well maintained. Only minimal inflammatory cell infiltration was detected in Fig. 2F, suggesting a robust histoprotective and anti-inflammatory effect of APME at this dosage.

### Immunohistochemical analysis

#### TNF-α expression

**Fig. 3:**
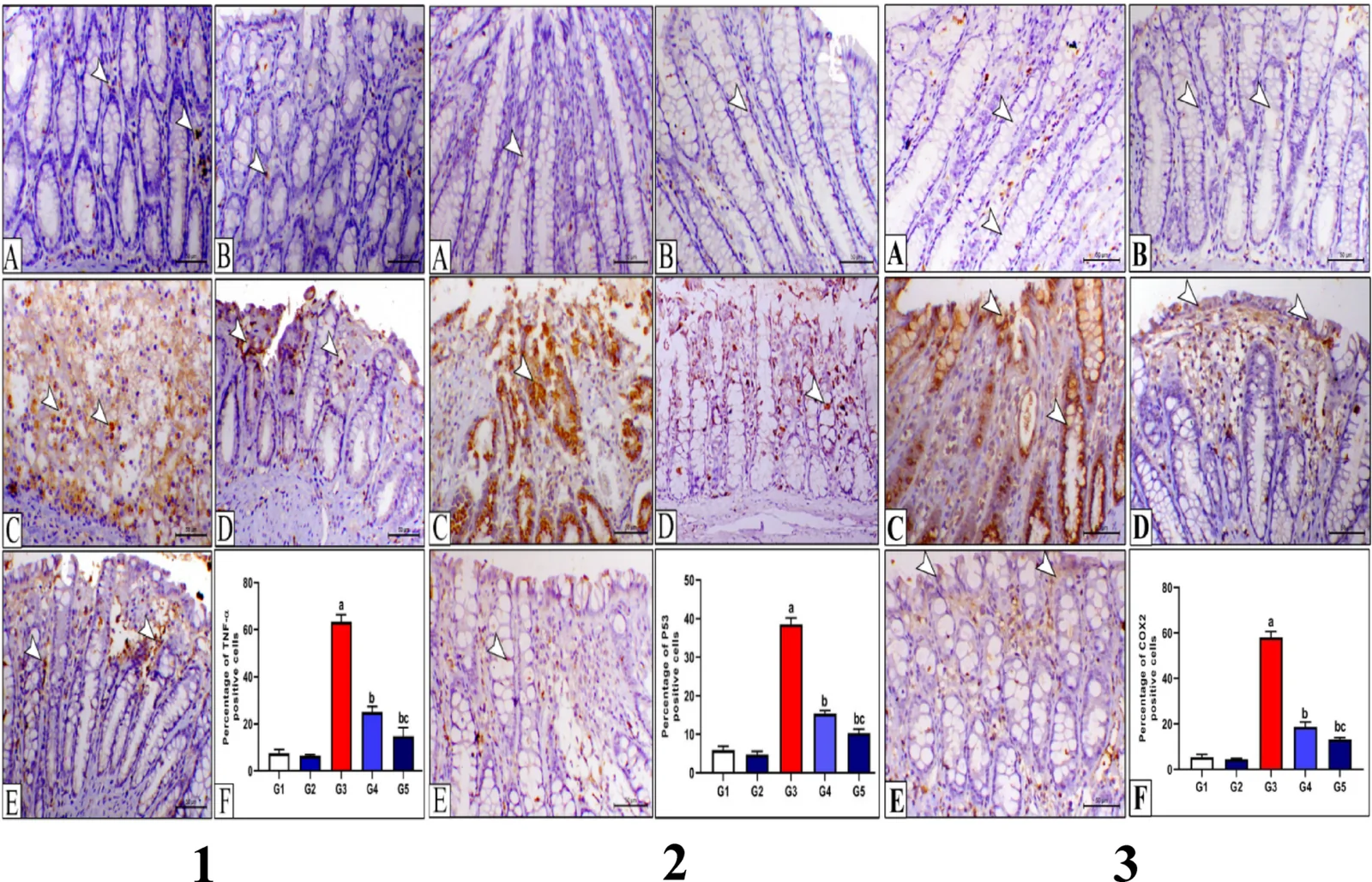
Immunohistochemical staining of inflammatory and stress markers [**1** TNF-α, **2** P53, and **3** COX-2] in the colonic mucosa across experimental groups (**A**–**E**), and quantitative analysis of marker-positive cells (**F**) (magnification × 200; scale bar = 50 μm). **1 TNF-α expression**; **A** Group 1 (control) and **B** Group 2 (200 mg/kg APME) display minimal TNF-α immunoreactivity (arrowheads). **C** Group 3 (ulcerative colitis) shows strong positive TNF-α expression (arrowheads), indicating a heightened inflammatory response. **D** Group 4 (100 mg/kg APME + colitis) exhibits moderate TNF-α immunoreactivity (arrowheads). **E** Group 5 (200 mg/kg APME + colitis) demonstrates reduced TNF-α expression, comparable to control levels (arrowheads). **F** Histogram showing the mean area percentage of TNF-α-positive cells in groups G1–G5. ^a^ *P* < 0.05 vs. Group 1; ^b^ *P* < 0.05 vs. Group 3; ^c^ *P* < 0.05 vs. Group 4. **2 P53 expression**; **A** Group 1 (control) and **B** Group 2 (200 mg/kg APME) exhibit minimal nuclear P53 immunoreactivity (arrowheads). **C** Group 3 (ulcerative colitis) reveals strong P53 expression (arrowheads), suggesting increased cellular stress and damage. **D** Group 4 (100 mg/kg APME + colitis) shows moderate P53 immunoreactivity (arrowheads). **E** Group 5 (200 mg/kg APME + colitis) displays reduced P53 expression with minimal staining (arrowheads), approaching baseline levels. **F** Histogram depicting the mean area percentage of P53-positive cells across G1–G5. ^a^ *P* < 0.05 vs. Group 1; ^b^ *P* < 0.05 vs. Group 3; ^c^ *P* < 0.05 vs. Group 5. **3 COX-2 expression**; **A** Group 1 (control) and **B** Group 2 (200 mg/kg APME) exhibit weak cytoplasmic COX-2 staining in a few epithelial cells (arrowheads). **C** Group 3 (Ulcerative colitis) shows intense COX-2 immunoreactivity in epithelial and lamina propria cells (arrowheads), indicating increased inflammatory activity. **D** Group 4 (100 mg/kg APME + colitis) reveals moderate COX-2 staining (arrowheads). **E** Group 5 (200 mg/kg APME + colitis) demonstrates COX-2 levels like the control (arrowheads), indicating inflammation resolution. **F** Histogram illustrating the mean area percentage of COX-2-positive cells in groupsG1–G5. ^a^ *P* < 0.05 vs. Group 1; ^b^ *P* < 0.05 vs. Group 3; ^c^ *P* < 0.05 vs. Group 5

Immunohistochemical staining for TNF-α revealed minimal expression in both the control group (G1) and the APME-only group (G2, 200 mg/kg), suggesting a baseline physiological level of this proinflammatory cytokine (Fig. 3(1)A&B). In contrast, colonic tissues from the ulcerative colitis group (G3) exhibited markedly intense TNF-α immunoreactivity, indicative of severe inflammation (Fig. 3(1)C).

Interestingly, animals in G4 (100 mg/kg APME + colitis) demonstrated moderate TNF-α staining (Fig. 3(1)D), indicating a partial attenuation of the inflammatory response. The most pronounced therapeutic effect was observed in G5 (200 mg/kg APME + colitis), where TNF-α expression was markedly reduced and comparable to the control Fig. 3(1)E, confirming the dose-dependent anti-inflammatory efficacy of APME.

#### p53 expression

Negligible p53 immunoreactivity was seen in both the control (G1) and APME-only (G2) groups, suggesting no genotoxic or apoptotic stress under normal conditions, Fig. 3(2)A&B. However, G3 (colitis group) exhibited intense p53 expression, consistent with epithelial DNA damage and stress-induced apoptotic signalling (Fig. 3(2)C).

Treatment with APME at 100 mg/kg (G4) resulted in a moderate decrease in p53 expression (Fig. 3(2)D), whereas the higher dose of 200 mg/kg (G5) restored p53 levels near baseline, like control levels (Fig. 3(2)E). This suggests that APME's antioxidant properties may mitigate oxidative DNA damage and inhibit apoptotic signalling via p53 downregulation.

#### COX-2 expression

Immunostaining for COX-2 in colon tissues from the control group (G1) and the APME-only group (G2; 200 mg/kg) demonstrated weak cytoplasmic immunoreactivity, localized mainly to scattered surface and glandular epithelial cells, reflecting basal expression levels of this inducible enzyme under non-inflammatory conditions Fig. 3(3)A&B.

In contrast, the ulcerative colitis group (G3), subjected to acetic acid instillation without treatment, displayed intense cytoplasmic COX-2 expression in both surface and glandular epithelium, consistent with severe mucosal inflammation and epithelial injury Fig. 3(3)C.

Interestingly, colonic sections from G4 (100 mg/kg APME + colitis) still exhibited elevated COX-2 immunoreactivity in numerous epithelial cells Fig. 3(3)D, suggesting that the lower dose of APME offered only partial anti-inflammatory protection. However, sections from G5 (200 mg/kg APME + colitis) revealed a marked reduction in COX-2 positive cells, Fig. 3(3)E, approaching control levels. This significant suppression highlights APME’s dose-dependent anti-inflammatory potential, likely mediated through attenuation of oxidative stress and modulation of the NF-κB/COX-2 axis.

### Morphometric analysis and statistical evaluation

Quantitative morphometric evaluation of the immunohistochemical staining revealed a significant elevation (*p* < 0.05) in the mean percentage area of positive immunoreactivity for TNF-α, P53, and COX-2 in the colitis-induced group (G3) when compared to the control group (G1). This sharp increase reflects the heightened inflammatory and apoptotic activity associated with acetic acid-induced ulcerative colitis.

Conversely, treatment with APME demonstrated a dose-dependent ameliorative effect. Both the low-dose treatment group (G4, 100 mg/kg b.w.) and the high-dose treatment group (G5, 200 mg/kg b.w.) showed a statistically significant reduction (*p* < 0.05) in TNF-α, P53, and COX-2 expression levels when compared to the colitis group (G3). Furthermore, a notable difference was observed between the two treated groups, with G5 exhibiting a more pronounced decrease in the expression of these inflammatory and apoptotic markers than G4.

These findings, supported by the graphical data presented in Fig. 3(1F, 2F, and 3F), underscore the potential of APME to downregulate inflammation and oxidative stress-induced cellular damage in a dose-responsive manner.

### Quantitative evaluation of APME’s anti-inflammatory and antioxidant effects in experimental UC

**Fig. 4:**
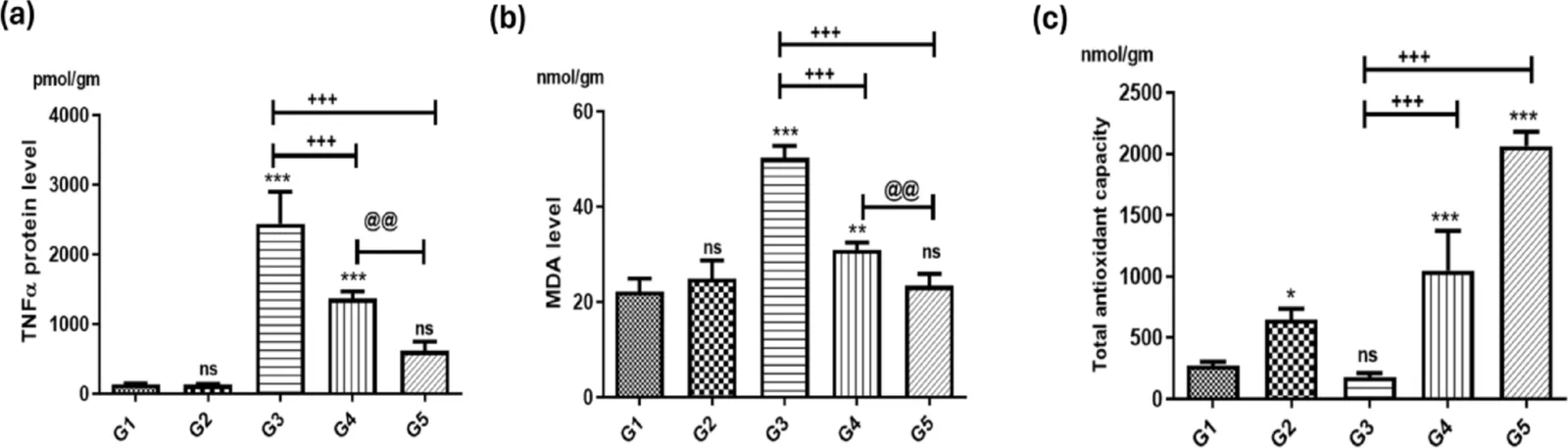
Assessment of proinflammatory and oxidative stress biomarkers **a** TNF-α via ELISA, **b** Malondialdehyde (MDA) and **c** Total antioxidant capacity via spectrophotometry. Data are expressed as mean ± SD. Statistical analysis was performed using one way ANOVA followed by Tukey’s multiple comparison test. (***) significance compared to the control group with *p* value < 0.05. (+ + +) significance of AMPE treated groups (G4, G5) compared to ulcerative colitis group (G3) with p value < 0.05. (@@) comparison of G5(200 mg/kg AMPE) Vs G4 (100 mg/kg AMPE) with p value < 0.05. ns means non-significant

The ulcerative colitis group (G3) demonstrated a significant increase in TNF-α gene expression (Fig. 4a) compared to the healthy control (G1), confirming the occurrence of acute mucosal inflammation. Conversely, treatment with APME resulted in a significant suppression of TNF-α expression (*p* < 0.05) in a dose-dependent manner compared to the ulcerative colitis group G3, with the higher dose showing a more pronounced anti-inflammatory response.

Regarding the oxidative stress marker, malondialdehyde (MDA) level was significantly abrogated in AMPE-treated groups 4 and 5 compared to colitis group 3 (Fig. 4b). The total antioxidant capacity (TAC) was significantly diminished in the colitis group (G3), indicating impaired redox homeostasis (Fig. 4c). Treatment with APME led to a substantial restoration of TAC in both treatment groups, with G5 achieving levels comparable to or exceeding those of the control group (G1), indicating enhanced antioxidant defense mechanisms.

**Fig. 5:**
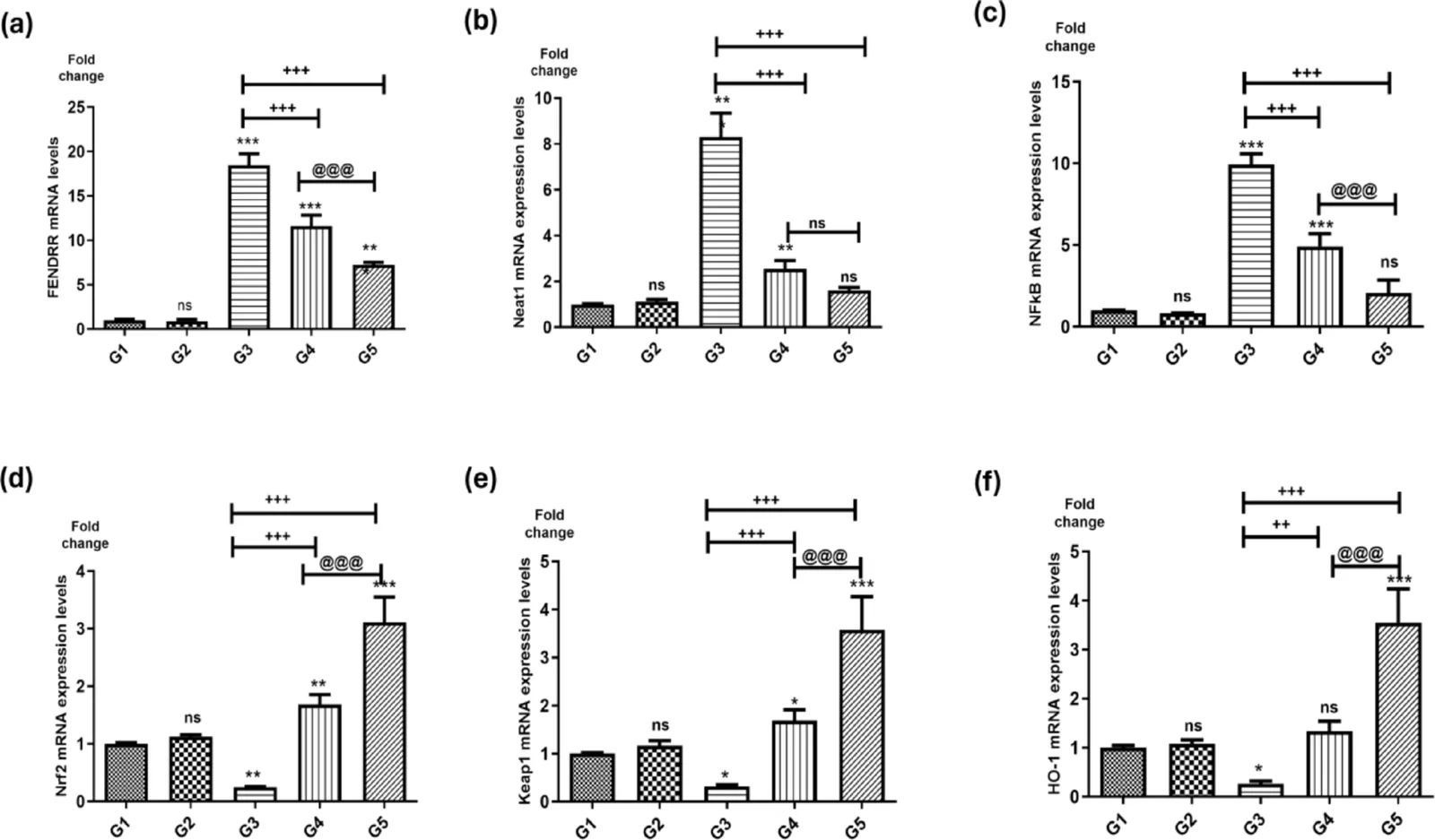
Quantitative PCR gene expression levels **a** FENDRR **b** Neat1 **c** NFκB **d** Nrf2 **e** Keap1 **f** HO-1. Data are expressed as mean ± SD. Statistical analysis was performed using one way ANOVA followed by Tukey’s multiple comparison test. (***) significance compared to the control group with p value < 0.05. (+ + +) significance of AMPE treated groups (G4, G5) compared to ulcerative colitis group (G3) with p value < 0.05. (@@) comparison of G5(200 mg/kg AMPE) Vs G4 (100 mg/kg AMPE) with *p* value < 0.05. ns means non-significant

Our data showed that HO-1 expression levels were significantly diminished in G3 (colitis group) compared to the control group. Treatment with AMPE restored the HO-1 gene expression in the treated groups (G4 and G5) compared to the colitis group in a dose-dependent manner (Fig. 5f). The Keap1/Nrf2 signalling pathway, comprising Nrf2 and its inhibitory counterpart, Kelch-like ECH-associated protein 1 (Keap1), has been shown to exert a protective effect in both experimental models and individuals with ulcerative colitis (Fig. 5d, e).

### Assessment of gene expression levels of FENDRR and Neat1 LncRNAs

The current findings demonstrate a marked upregulation of both FENDRR and Neat1 in G3, followed by a progressive downregulation in G4 and G5, suggesting a disease-associated elevation with subsequent modulation by treatment (Fig. 5a, b).

#### FENDRR expression

In our study, FENDRR was significantly elevated in G3 (18.44 ± 1.00), indicating strong transcriptional activation, likely in response to inflammatory or fibrotic signaling pathways. The observed mitigation of FENDRR levels in groups 4 and 5 (11.619 ± 1.2 and 7.25 ± 0.289, respectively) likely reflects the intervention’s capacity to reduce inflammation-mediated fibrotic gene expression.

#### NEAT1 expression

Similarly, NEAT1 expression peaked in GP 3 (8.3 ± 1.054), supporting its well-documented role in inflammation and immune regulation (Liu et al. 2018). Notably, the downregulation observed in GPs 4 and 5 (2.546 ± 0.362 and 1.6 ± 0.135, respectively) indicates a potential anti-inflammatory effect.

## Discussion

AP is a halophytic perennial member of the genus* Atriplex* with restricted phytochemical and biological investigations (Zanella and Vianello 2020; Hamada et al. 2025). Despite Abd El Raheim (2013) reporting that pretreatment with 400 mg/kg extracts of *Atriplex farinosa* and or *Atriplex nummularia* effectively protected against colonic ulceration and attenuated the elevation of myeloperoxidase activity, no anti-UC study was reported at AP.

Bioactive phytoconstituents contribute significantly to the maintenance of human health and alleviate organ malfunction (Davila and Papada 2023). Antibacterial organic acid may support the intestinal barrier integrity (Raybaudi-Massilia et al. 2009; Ricciutelli et al. 2020; Mu et al. 2012; Fukahori et al. 1996).

Caffeic acid is a common hydroxycinnamic acid found in many fruits. It showed a significant role in modulating intestinal inflammation by reducing COX-2 expression, PGE2 production, and IL-8 biosynthesis in IL-1β-treated human colonic myofibroblasts, while also inhibiting advanced glycation end product formation through strong chelating activity (Zieli´nska et al. 2021).

Flavonoids possess considerable therapeutic value in the management of inflammatory diseases due to their ability to inhibit various pro-inflammatory proteins, thereby functioning as natural modulators and reducers of inflammation`s severity (García-Lafuente et al. 2009). Beyond their direct antioxidant activity, flavonoids also regulate the expression of numerous antioxidants and cytoprotective genes through modulation of nuclear transcription factors and suppression of key inflammatory pathways (Lu et al. 2013). Furthermore, flavonoids positively influence gut microbiota composition by promoting the growth of beneficial bacteria such as *Bifidobacterium* and *Lactobacillus* species, thus contributing to an anti-inflammatory intestinal environment (Duda-Chodak et al. 2015; Parkar et al. 2013; Cardona et al. 2013; Satokari 2020). According to Formiga (2020), the co-administration of rosmarinic acid and *p*-cymene demonstrated notable anti-inflammatory activity within the intestinal tract.

Meanwhile, the essential oil of *Bunium persicum* decreased acetic acid-induced rat colitis via suppression of the NF-κB pathway (Rashidian et al. 2021).

In the context of IBD treatments, stigmasterol exhibits notable anti-inflammatory effects by modulating gut microbiota, enhancing butyrate production, suppressing key cytokines via NF-κB inhibition, strengthening the mucosal immunity, and intestinal barrier function. Moreover, 20-hydroxyecdysone showed antioxidant and anti-inflammatory activities (Wen et al. 2021; Jie et al. 2022; Bakrim et al. 2022; Dinan et al. 2021).

Research indicates that excessive dietary sugar intake alters gut microbiota composition by increasing the relative abundance of *Proteobacteria* while reducing *Bacteroidetes*, a phylum known to contribute to endotoxin neutralization and maintenance of gut barrier integrity. This imbalance shifts the microbial profile toward a pro-inflammatory state and impairs the regulation of epithelial and mucosal immune functions. As a result, high sugar consumption can drive metabolic endotoxemia and low-grade systemic inflammation, ultimately contributing to metabolic dysfunction. These findings suggest that, beyond supplying surplus energy, high dietary sugar may exert multiple adverse effects on health through microbiota-mediated pathways (Khakh and North 2012). Despite this evidence, Zhang (2022) found that sucrose reduces inflammatory cytokine expression only at low doses.

Our data on AP`s total phenolic and flavonoid contents are in harmony with those reported by Zanella & Vianello (2020). Among the thirty-five tentatively detected APME`s phytoconstituents [Table S1], organic acids [D-(+)-malic acid **1**, 2-isopropylmalic acid **2**, D-3-Phenyllactic acid **3, p**-hydroxybenzoic acid **4**, 2,5-dihydroxybenzoic acid **5],** phenolic acids [caffeic acid **6** & rosmarinic acid **7**], flavonoid glycosides [myricetin **8,** baicalein-7-*O*-glucuronide **9**, kaempferol-3-*O*-*α*-L-rhamnoside **10**, isorhamnetin-3-*O*-rutinoside **11,** okanin-4’-*O*-glucoside **12**, kaempferol-7-O-neohesperidoside **13**, kaempferol-3-Glucuronide **14,** 3,5, 7-trihydroxy-4’-methoxyflavone **15**] procyanidin B2 **16**, gluconate **18,** UDP-xylose **27** and γ-terpinene are likely the key contributors to its observed anti-UC efficacy. We propose that their synergistic interaction may offset the adverse impact of its carbohydrate content.

Furthermore, two effective anti- IBD metabolites [stigmasterol **1** and 20-hydroxyecdysone **2**] were isolated from APME`s fractions. To date, there is no definitive evidence that stigmasterol has been previously isolated from AP, although 20-hydroxyecdysone has been reported from its roots (Ben Nejma et al. 2015). These findings provide a strong incentive for exploring APME`s effectiveness in UC therapy through an in vivo study.

During our study of the survival group G2, it is reasonable to state that there is no evidence of AP`s toxicity as documented by Hamada (2025) and Abd El Raheim (2013).

Histological assessment of hematoxylin and eosin (H&E)-stained sections from UC`s patients showed a marked increase in the apoptosis rate of colonic mucosal epithelial cells. This heightened cell turnover, characterized by excessive apoptosis and compensatory proliferation, contributes to the structural disruption of crypt architecture and compromises the integrity of the intestinal mucosal barrier (Zhao et al. 2016). In renal fibrosis, Huangqi decoction demonstrated a dose-dependent protective effect on the ipsilateral kidney by suppressing the expression of TGF-β1, TGF-β receptors I and II, Smad2, phosphorylated Smad2 (P-Smad2), Smad4, α-SMA, and collagens I, III, and IV. Conversely, it enhanced the expression of the inhibitory regulator Smad7 (Luo et al. 2022). Likewise, our histological analysis of the tested groups confirmed APME's safety, ability to reduce induced inflammation and cytoprotective efficacy (Fig. 2A–F).

Colitis induction resulted in a marked elevation of proinflammatory mediators, including TNF-α, PTX3, and PAF in plasma, as well as increased TLR4 expression in colonic tissue (Ercan et al. 2025). Phytochemicals capable of downregulating TNF-α secretion or interfering with its associated inflammatory signalling pathways may serve as promising alternatives for the management of immune-mediated inflammatory disorders (Subedi et al. 2020).

Overexpression of the p53 protein in colorectal crypts is frequently observed in ulcerative colitis (UC) patients, even in the absence of histopathological evidence of dysplasia. This overexpression is often interpreted by pathologists as an intermediate state between regenerative epithelial changes and intraepithelial neoplasia, and it serves as a valuable biomarker for assessing the risk of malignant transformation (Popp et al. 2016; Rubin and Turner 2006). Notably, a high frequency of p53 mutations has been reported in individuals with long-standing, severe UC who have not yet developed colorectal cancer, highlighting the role of chronic inflammation in driving early molecular alterations associated with carcinogenesis (Itzkowitz 2003; Hussain et al. 2000).

In the study conducted by Sukumari (2011), certain clinically relevant dietary phytochemicals significantly suppressed the proliferation of human S-type neuroblastoma (NB) cells. Notably, these antiproliferative effects appeared to be functionally independent of both caspase and p53 activation and were instead associated with inhibition of the Akt-NFκB signalling pathway (Sukumari-Ramesh et al. 2011).

Although COX-2 expression is generally low in the GI of healthy humans and animals, it plays a pivotal role in maintaining mucosal defence mechanisms. COX-2 is essential for promoting the resolution of inflammation, facilitating ulcer healing, and mediating long-term adaptive changes in GI function following inflammatory episodes (Wallace and Devchand 2005). The phytochemicals identified by Desai (2018) have demonstrated significant inhibitory effects on cyclooxygenase-2 (COX-2), an enzyme commonly upregulated during inflammatory processes and tumorigenesis through cancer-associated signalling pathways (Desai et al. 2018). Similarly, combined administration of both methanolic extracts from Egyptian propolis and *Moringa oleifera* Lam. (Moringaceae) exhibited a protective effect against acetic acid-induced ulcerative colitis in rats. Both synergistically inhibited COX-1 and COX-2 activity, lowered the ulcerative index and lesion scores, and attenuated oxidative stress markers and pro-inflammatory mediators (Atta et al. 2019). These findings are consistent with previous studies demonstrating that polyphenol and flavonoid rich plant extracts reduce COX-2 expression and inflammatory mediators in chemically induced colitis models (Salaritabar et al. 2017). The ability of APME to diminish COX-2 overexpression reinforces its therapeutic value in managing UC through modulation of inflammatory enzyme pathways.

Importantly, APME remedies noticed markedly reduced TNF-α, p53, and Cox-2 expression levels in a dose-dependent manner (Fig. 3(1, 2 & 3(A–F))).

Lipid peroxidation, reflected by MDA levels, was significantly attenuated in AMPE-treated groups 4 and 5 compared to colitis group 3 (Fig. 4b). This indicates the efficacy of AMPE in the amelioration of oxidative stress induced by inflammation. On the other hand, total antioxidant capacity (TAC) was significantly diminished in the colitis group (G3), indicating impaired redox homeostasis (Fig. 4c). Treatment with APME led to a substantial restoration of TAC in both treatment groups, with G5 achieving levels comparable to or exceeding those of the control group (G1), indicating enhanced antioxidant defense mechanisms. These line with that nuclear factor erythroid 2–related factor 2 (Nrf2) serves as a key transcriptional regulator responsible for maintaining redox balance and activating cellular antioxidant defences (He et al. 2020).

The upregulated levels of TNF-*α* in the colitis group resulted from the deregulation of NFκB signalling induced by concomitant oxidative stress (Wang et al. 2016). Our data revealed significant upregulation of NFκB gene expression in the G3 colitis group, which is significantly counteracted by treatment with AMPE in treated groups (G4, G5) in a dose-dependent manner (Fig. 5c). In the same context, Zahedipour (2022) highlighted the downregulating effect of several phytochemicals on TNF-α in inflammatory conditions.

Hemeoxygenase-1 (HO-1) is a downstream target of Nr2 (Alsharif et al. 2022). Our data showed that HO-1 expression levels were significantly diminished in G3 (colitis group) compared to the control group. Treatment with AMPE restored the HO-1 gene expression in the treated groups (G4 and G5) compared to the colitis group in a dose-dependent manner (Fig. 5f). The Keap1/Nrf2 signalling pathway, comprising Nrf2 and its inhibitory counterpart, Kelch-like ECH-associated protein 1 (Keap1), has been shown to exert a protective effect in both experimental models and individuals with ulcerative colitis (Fig. 5d,e). This pathway is thus essential for the antioxidant response and intestinal protection (Piotrowska et al. 2021).

Collectively, these findings suggest that APME exerts potent anti-inflammatory effects by inhibiting TNF-α expression, while concurrently restoring antioxidant capacity in colonic tissues. This dual action supports APME's potential as a complementary therapeutic agent in managing ulcerative colitis by targeting both inflammatory cytokines and oxidative stress.

For instance, Adylova (2021) reported that FENDRR expression was markedly elevated in colonic tissues of mice with DSS-induced colitis and was associated with enhanced TGF-β/Smad signaling activity. Similarly, Sun and Kraus (2015) found FENDRR upregulation in models of cardiac and pulmonary fibrosis, supporting its role as a pro-fibrotic LncRNA. The decline in FENDRR expressions in GPs 4 and 5 after treatment may reflect the therapeutic effect of the intervention in suppressing inflammation-induced fibrotic gene expression, consistent with findings from Lee (2022) who observed that pharmacological inhibition or genetic knockdown of FENDRR reduced fibrosis and inflammation in experimental colitis models.

lncRNAs NEAT1 are involved in the underlying mechanisms of UC. Studies using IBD mouse models have demonstrated that suppression of NEAT1 expression attenuates the inflammatory response, enhances intestinal barrier function, and modulates macrophage polarization toward an anti-inflammatory phenotype. Elevated NEAT1 expression has been associated with impaired intestinal barrier integrity in IBD, contributing to disease progression and mucosal dysfunction. The elevated NEAT1 expression in our study mirrors this pattern, suggesting its contribution to colonic inflammation and tissue damage (Liu et al. 2018).

Liu (2018) documented that NEAT1 expression is significantly upregulated in a murine model of IBD, where it contributes to the modulation of intestinal inflammation through exosome-mediated regulation of macrophage polarization. Notably, silencing NEAT1 alleviated intestinal inflammation in mice; however, the precise molecular mechanisms underlying NEAT1’s role in intestinal immune regulation remain incompletely understood.

Overall, the expression trends of FENDRR and NEAT1 in this study are consistent with existing literature that links these lncRNAs to inflammatory and fibrotic responses in colonic and systemic disease models. The observed reduction in expression following treatment supports their role as biomarkers of disease activity and potential targets for therapeutic modulation.

## Conclusion

AP is a halophytic plant with variant bioactive phytochemicals (Zanella and Vianello 2020), yet its anti-UC impact remains unexplored in animal models. APME was assessed for its total phenolic and flavonoid contents. Negative ionization mode LC–ESI–MS/MS analysis of APME revealed 35 bioactive metabolites. Additionally, stigmasterol **1** and 20-hydroxyecdysone **2** were isolated from the *n*-hexane and ethyl acetate fractions, respectively, using column chromatography followed by spectroscopic characterization. APME demonstrates promising antioxidant and anti-inflammatory properties in a dose-responsive pattern. It ameliorates histological damage, reduces inflammatory cytokine production, and restores oxidative balance. These effects are mediated by modulation of transcription factors (Nrf2, NF-κB), inflammatory mediators (TNF-α, COX-2, P53), and regulatory lncRNAs (FENDRR, Neat1). Although these findings support the therapeutic potential of APME, further investigations are warranted, including clinical trials to establish its efficacy.

## Supplementary Information

Below is the link to the electronic supplementary material.

Supplementary Material 1.

## Data Availability

All data generated or analyzed during this study are included in this published article and its supplementary information files. Additional details are available from the corresponding author upon reasonable request.
