# Supplementary material for: Atriplex portulacoides L. derived phytochemicals mitigate acetic acid induced colitis in rats via orchestrating Nrf2/Keap1 signalling and LncRNAs gene expression
Source: Inflammopharmacology. 2026 Jul 30;34(9):6275–89. doi: 10.1007/s10787-026-02332-0 (PMC13558346; doi:10.1007/s10787-026-02332-0)
Supplement: Supplementary file 1 [file 10787_2026_2332_MOESM1_ESM.docx]

**Supplementary materials**

***Atriplex portulacoides* L*.* derived phytochemicals mitigate acetic acid induced colitis in rats via orchestrating Nrf2/Keap1 signalling and LncRNAs gene expression.**

Maha Hassan^a^, Marwa M. Mona^b^, Marwa M. Abd-Elsalam^c^, Mona El-Aasr^a^, Amal Kabbash^a^ & Ghada Attia^a*^

*^a^* Postgraduate Master student (Pharmacognosy Department), Faculty of Pharmacy, Tanta University, Tanta (31527), Egypt.

*^a^* Department of Pharmacognosy, Faculty of Pharmacy, Tanta University, Tanta (31527), Egypt.

*^b^* Medical Biochemistry and Molecular Biology Department, Faculty of Medicine, Kafrelsheikh University, Kafrelsheikh (33511**)**, Egypt

*^c^* Histology and Cell Biology, Faculty of Medicine, Kafrelsheikh University, Kafrelsheikh (33511**)**, Egypt

^*^ Corresponding author email: [ghada.attia@pharm.tanta.edu.eg](mailto:ghada.attia@pharm.tanta.edu.eg)

**Materials and methods**

**Instruments and reagents for APME chromatographic and biological studies**

For qualitative and quantitative analysis of APME’s phytoconstituents, GC/MS Finnigan Mat. SSQ7000, Trace GC 200 (thermo) GC mode, USA. ELISA Processor II Microplate Reader (EXL800, USA.). Jasco, FT/IR-6100, Japan. Q-TOF-LC/HRMS system, 6530 (Agilent Technologies) equipped with an autosampler (G7129A) with an ESI ionization source (USA).

For isolation and identification of metabolites from APME’s fractions, Shimadzu UV spectrophotometer UV- 1800, Japan. Bruker Avance III spectrometer (Germany) with 400 MHz for ^1^H NMR, 100 MHz for ^13^C NMR. ESI- MS spectra (CMS from Advion Co., NY, USA). Rotary evaporator (IKA RV10 D599, Wilmington, North Carolina, USA. Analytical balance Sartorius type 2842, Germany. Digital balance, Scout TM Pro (OHAUS) model (PA413, Ohaus Corp., USA). Water deionizer, Puris-ExpeUPEle UV-M, South Korea. Thermostatic oven (WT-binder 7200), Germany. Glass columns of different sizes. Precoated TLC silica gel sheets G F254 (E. Merck, Germany). Chemicals for phytochemical analysis included HPLC-grade methanol (≥99.9%) purchased from Sigma Co. (St. Louis, USA), ferric chloride, sodium carbonate, gallic acid, AlCl₃, sodium acetate, rutin, and Folin-Ciocalteu reagent (manufacturer details needed) for total phenolic content detection. Solvents for extraction were of analytical grade HPLC grade ≥ 99.9 %, purchased from Sigma Co. (St. Louis, USA); methanol, *n*-hexane (65-70°C), methylene chloride, ethyl acetate, *n*-butanol, and acetic acid. Spry reagents for TLC studies, including alcoholic *α*-naphthol, sulphuric acid, glacial acetic acid, acetic anhydride, and sodium hydroxide, which were prepared according to Stahl (1962).

For biological analysis, a pediatric propylene Foley catheter (size 8 Fr, Romsons, India), a Leica digital camera (ICC50; Switzerland), a Leica light microscope (DM500, Switzerland), a centrifuge (Sigma laborzenrifugen D-37520 Osterode am Harz, Germany), and a Stratagene MX3005P real-time PCR machine (manufacturer details needed) were used.

Additional chemicals for biological studies included isoflurane, saline, K-Y jelly, methyl cellulose, 3% acetic acid, EDTA, sodium pentobarbital, Rat-TNF-α ELISA kit, cat no: ER1393 (Fine Test, Wuhan Fine Biotech Co, China). RLT buffer (RNA safeguard, Bioflux), *β*-mercaptoethanol, QIAamp RNeasy Mini Kit (Qiagen, Germany, GmbH), ethanol, oligonucleotide primers (Metabion, Germany), 2x QuantiTect SYBR Green PCR Master Mix (Qiagen, Germany, GmbH), and RevertAid Reverse Transcriptase (200 U/µL, Thermo Fisher), Rat-TNF-α ELISA kit cat no: ER1393 (Fine Test, Wuhan Fine Biotech Co, China). Malonaldehyde (MDA) by OxiSelect™ TBARS Assay Kit, Cell Biolabs, San Diego, USA. OxiSelect™ total antioxidant capacity (TAC) assay Kit, Cell Biolabs, San Diego, USA, hematoxylin & eosin (H&E) (Bancroft, J.D, and Layton, C.,2010), formalin, paraffin, and xylene.

For antioxidant and redox assays, the following kits were used: OxiSelect™ TBARS Assay Kit for malondialdehyde (MDA) measurement and OxiSelect™ Total Antioxidant Capacity (TAC) Assay Kit (both from Cell Biolabs, San Diego, USA). All biochemical reagents were of analytical grade and sourced from Sigma-Aldrich.

Cell-based investigations were supported using a Stratagene Mx3005P real-time PCR system (Agilent Technologies, USA), along with a centrifuge (Hermle Z206A, Germany). Total RNA was extracted using RLT buffer (RNA safeguard, Bioflux, Japan) and the QIAamp RNeasy Mini Kit (Qiagen GmbH, Germany), with β-mercaptoethanol and ethanol (≥99.9%, Sigma-Aldrich) used as supporting reagents. Complementary DNA synthesis and gene amplification were carried out using RevertAid Reverse Transcriptase (200 U/μL, Thermo Fisher Scientific, USA), 2x QuantiTect SYBR Green PCR Master Mix (Qiagen GmbH, Germany), and custom oligonucleotide primers (Metabion, Germany).

Histological assessment involved formalin fixation, paraffin embedding, and sectioning followed by hematoxylin and eosin (H&E) staining, prepared according to standard protocols (Bancroft & Layton 2010). Imaging and microscopy were performed using a Leica DM500 light microscope and a Leica ICC50 digital camera (Leica Microsystems, Switzerland) at the Histology and Cell Biology Department, Faculty of Medicine, Kafrelsheikh University.

**Total phenolics & total flavonoids examination of APME**

The total phenolic content was determined using the Folin–Ciocalteu assay, following the protocol described by Attard (2013). 1mg/ml of gallic acid alcoholic stock solution was prepared and serially diluted into concentrations of 250, 125, 62.5, 31.2, 15.6, and 7.8 μg/ml. The sample alcoholic solution was prepared at a concentration of 3 mg/ml. The procedure involved combining 10 μL of sample or standard with 100 μL of Folin–Ciocalteu reagent (diluted 1:10) in a 96-well microplate. 80μL of 1M sodium carbonate was added and incubated at room temperature (25 ͦ C) for 20 min in the dark. The resultant blue complex color was measured at 630nm by the FluoStar Omega microplate reader. Finally, data are represented as means ±SD.

The total flavonoid content was assessed using the aluminium chloride method, as outlined by Kiranmai (2011). A gallic acid stock solution of 1 mg/ml in methanol was prepared, where seven serial dilutions were made in concentrations of 500, 250, 125, 62.5, 31.2, 15.6, and 7.8 μg/ml for total phenolics determination. A stock solution of 1 mg/ml in methanol was prepared, and six serial dilutions were made in the concentrations of 1000, 500, 250, 150, 100, and 50 μg/ml. APME samples were prepared at a concentration of 3 mg/ml in ethanol. An aliquot of 15 μl of sample or standard was dispensed into a 96-well microplate, followed by the addition of 175 μl of methanol, 30 μl of 1.25% aluminum chloride, and 30 μl of 0.125 M sodium acetate. The mixture was incubated for 5 minutes, where the resulting yellow colour was measured at 510 nm by using a microplate reader, FluoStar Omega. Data were represented as means ±SD.

**LC-ESI-MS /MS analysis of APME**

The chemical profiling of APME was performed at the Proteomics and Metabolomics Unit, Basic Research Department, Children’s Cancer Hospital (57357, Cairo, Egypt) according to the literature (Mohammed et al. 2021) Secondary metabolites were analyzed using a high-flow liquid chromatography system (ExionLC, Sciex®, Framingham, MA, USA) coupled with a TripleTOF® 5600+ mass spectrometer (Sciex®), operated in information-dependent acquisition (IDA) mode. System control and data acquisition were carried out using Analyst® TF software version 1.7.1 (Sciex®).

The APME sample (50g/ml) was filtered through in-line filter discs (0.5 µm × 3.0 mm, Phenomenex®, Torrance, CA, USA) before injection into an XSelect® HSS T3 XP UHPLC column (2.5 µm, 2.1 × 150 mm, Waters®, Milford, MA, USA) maintained at 40 °C. Chromatographic separation was achieved using two mobile phases: solvent A (1% methanol in 5 mM ammonium formate buffer, pH 8) and solvent B (100% acetonitrile), delivered at a flow rate of 0.3 mL/min. A gradient elution was applied, starting with 90% solvent A or B and 10% solvent C for 20 minutes, followed by a switch to 10% solvent A or B and 90% solvent C for 5 minutes, and finalized with a return to the initial composition for the last 3 minutes.

Total ion chromatograms (TICs) were used to extract peaks based on a signal-to-noise ratio greater than 5 and a minimum of threefold intensity difference between sample and blank (non-targeted analysis). Data interpretation involved converting Wiff files to ABF format using the Reifycs ABF Converter (Reifycs®, Tokyo, Japan), followed by data processing and feature alignment using MS-DIAL version 4.6 (RIKEN®, Tokyo, Japan). Peak identification was further confirmed using PeakView™ software version 21 (Sciex®), which compared retention times and m/z values obtained from MS and MS² spectra. Extracted ion chromatograms (XICs) were automatically generated and quantified using the XIC Manager tool, based on user-defined intensity thresholds.

**Chromatographic separation and spectroscopic identification of metabolites from APME’s fractions:**

A total of 2.8 kg of dried aerial parts of *Atriplex portulacoides* L. were finely ground and subjected to cold maceration with methanol. APME was concentrated under reduced pressure using a rotary evaporator, yielding 120 g of crude methanolic extract. The APME was suspended in water and successively fractionated with different organic solvents, then concentrated by rotary evaporator. The fractions obtained were *n*-hexane (6.54 g), methylene chloride (8.44 g), ethyl acetate (7.51 g), and *n*-butanol (27 g), respectively. These fractions were subjected to column chromatography for the isolation of pure metabolites. Compounds were successfully isolated from *n*-hexane and ethyl acetate fractions, whereas the remaining fractions require further studies to obtain a distinct pure metabolite.

A portion of the n-hexane fraction (6 g) was subjected to saponification following the method described by El Said and Amer (1965). The resulting mixture was separated with appropriate solvents and concentrated under reduced pressure, yielding 0.8 g of the saponified fraction (SAP) and 1.146 g of the unsaponifiable fraction (USAP). The unsaponifiable residue (USAP) was further purified via column chromatographic techniques on silica gel (*ϕ* 1 × 50 cm, 30 g). A gradient elution was employed, beginning with *n*-hexane and gradually increasing polarity using methylene chloride, followed by methanol. Fractions of 30 mL each were collected and analysed by TLC on silica gel G sheets, using solvent systems methylene chloride-methanol (9:1) and methylene chloride-methanol (8:2), and sulfuric acid spray for visualization. Fractions exhibiting similar TLC profiles were combined and concentrated under reduced pressure, yielding fourteen distinct groups. Twenty-five milligrams were obtained from the pooled fractions no. 80–84 of group VI, eluted with *n*-hexane:methylene chloride (20:80). TLC analysis using the solvent system methylene chloride-methanol (8:2) revealed a single dark brown spot. Upon repeated crystallization from methanol, a white powder designated as compound **1** (20 mg) was isolated, with *R_f_* = 0.64 at solvent system hexane: methylene chloride (20:80).

The ethyl acetate-soluble fraction (6.5 g) was subjected to chromatography on a silica gel column (*ϕ* 4 x 32 cm, 195 g). The metabolites were gradient-eluted from the column using n-hexane, then gradually increasing polarity with ethyl acetate to produce eleven groups. **Group III** [283 mg; containing fractions (295-314), which eluted with ethyl acetate: methanol (93: 7)] was TLC analysed by solvent system methylene chloride-methanol-water (8:2:0.2) to reveal a minor green spot and an intermediate-intensity brown one with an R*_f_* of 0.57 and 0.80, respectively. This group was further chromatographed on silica gel (*ϕ* 0.5 × 16.5cm, 8.5g), where elution was initiated with pure methylene chloride (100%), followed by a stepwise increase in polarity using solvent mixtures of methylene chloride: methanol: water in the ratios 9:1:0.1, 8:2:0.2, 7:3:0.5, and 6:4:0.5. This yielded five sub-fractions III a–e. **Group III c** (10 mg) eluted with methylene chloride: methanol: water (8:2:0.2) was crystallized from 100% methanol by methylene chloride. This led to the isolation of compound **2** (8 mg), a yellowish amorphous solid. Silica gel TLC analysis of compound **2** gave a dark brown spot upon heating with 10% H_2_SO_4,_ with *R_f_* value = 0.77 at solvent system methylene chloride: methanol: water (8:2:0.2).

Different spectroscopic techniques structurally elucidated these two pure compounds. The IR, ^1^H NMR, ^13^C NMR, and ESI-MS spectral data for compounds **1** and **2** were consistent with those previously reported in the literature (Jain & Bari 2010; El-Shial et al. 2023; Chaturvedula & Prakash 2012; Ben Nejma et al. 2015)

**Compound 1** (stigmasterol; C_29_H_48_O) an amorphous white powder. IR (KBr) *vmax*: 3414.30, 2924.73, 2868, 2855.78, 1621.87, 1462.02, 1383.43, 1052.58, and 618.94 cm^-1^. 1H-NMR (400 MHz, CD_3_OD-*d4*) *δ_H_* ppm; 3.56 (m, 1H, H-3,), 5.38 (m, 1H, H--6*,* d), 0.71 (s, 3H, H-18), 1.03 (s, 3H, H-19), 0.95 (d*, J* = 6 Hz, 3H, H-21), 5.07(m, 3H, H-22), 5.19 (m, 3H, H-23), 0.81 (d*, J* = 6.7 Hz, 3H, H-26), 0.84 (d*, J* = 6.8 Hz, 3H, H-27), 0.87 (d*, J* = 8 Hz, 3H, H-29), 0.67-2.3 (m, nH, H-1, 2,4,7-9, 11, 12, 14-17, 20, 24, 25 and 28). ^13^CNMR (100MHz, CD3OD-*d4*) *δ_C_* ppm: 37.3 (CH_2_, C-1), 31.9 (CH_2_, C-2), 71.8 (CH, C-3), 42.3 (CH_2_, C-4), 140.8 (C, C-5), 121.7 (CH, C-6), 30.5 (CH_2_, C-7), 30.5 (CH, C-8), 50.2 (CH,C-9), 35.1 (C, C-10), 20.9 (CH_2_, C-11), 39.8 (CH_2_,C-12), 42.3 (C, C-13), 56.8 (CH, C-14), 23.1 (CH_2_, C-15), 27.1 (CH_2_, C-16) , 56.2 (CH, C-17), 12.0 (CH_3_, C-18), 19.1 (CH_3_,C-19), 40.7 (CH, C-20), 21.1 (CH_3_, C-21), 138.7 (CH, C-22) , 129.0 (CH, C-23), 45.9 (CH, C-24), 29.1(CH, C-25), 19.4 (CH_3_, C-26), 19.8 (CH_3_, C-27), 25.2 (CH_2_,C-28), 11.9 (CH_3_, C-29). ESI-MS protonated molecular ion peak at 413.1 *m/z*_._

**Compound 2** (20-hydroxyecdyson; C_27_H_44_O_7_) a yellowish amorphous solid. IR (KBr) *vmax*: 3739.01,3418.08, 2922.84^,^ 2855.24, 1633.40, 1458.41, 1381.68, 1030.12, and 615.48 cm^˗1^. ^1^H-NMR spectral data (DMSO-d_6_, 400 MHz) *δ_H_* ppm: 3.81 (m, 1H, H-2), 3.92 (m, 1H, H-3), 2.28(m, 1H, H-5), 5.40 (s, 2H,H-7), 3.20 (m, 1H, H-9), 2.42 (M, 1H,H-17), 0.86 (s, 3H,H-18), 0.99 (s, 3H,H-19), 1.26 (s, 3H,H-21), 3.35(m, 1H, H-22), 1.08(s, 6H,H-23 and H-26). ^13^C-NMR spectral data (DMSO-d_6_, 100 MHz) *δ_C_* ppm: 37.1 (CH_2_, C-1), 67.1 (CH, C-2), 67.3 (CH, C-3), 31.9 (CH_2_, C-4), 50.6 (CH, C-5), 217.3 (Q.C., C-6), 120.9 (CH, C-7), 166.9 (C, C-8), 33.7 (CH, C-9), 38.1 (C, C-10), 23.2 (CH_2_, C-11), 31.3 (CH_2_, C-12), 48.3 (C, C-13), 83.5 (C, C-14), 30.8 (CH_2_, C-15), 20.3 (CH_2_, C-16), 48.4 (CH, C-17), 16.7 (CH_3_,C-18), 20.5 (CH_3_, C-19), 76.6 (C, C-20), 17.6 (CH_3_, C-21), 76.7 (CH, C-22), 26.5 (CH_2_, C-23), 41.8 (CH_2_, C-24), 69.2 (C, C-25), 27.7 (CH_3_, C-26), 28.9 (CH_3_, C-27). ESI-MS deprotonated molecular ion peak at 479.1 *m/z*_._

**Materials for APME Biological Study**

**Induction of UC**

Acetic acid (100%) was procured from El Nasr Pharmaceutical Chemicals Company (Lot No. A0198111, Oubour, Qalyubia, Egypt). To prepare a 4% solution, 40 mL of concentrated acetic acid was diluted with 60 mL of distilled water.
Before the induction of colitis, rats were allowed a one-week acclimatization period under standard laboratory conditions. Twenty-four hours before colitis induction, animals were fasted but had free access to water. Under isoflurane inhalation anesthesia, a pediatric propylene Foley catheter (size 8 Fr) was gently inserted into the rectum to a depth of approximately 8 cm following lubrication with K-Y Jelly. Subsequently, 2 mL of 4% acetic acid was carefully instilled into the colon and retained for approximately 30 seconds to minimize backflow and ensure proper mucosal exposure, following established protocols for chemically induced ulcerative colitis models (Millar et al. 1996).

**Immunohistochemical study**

Formalin-fixed, paraffin-embedded colonic tissue sections were used for immunohistochemical analysis, employing the avidin–biotin peroxidase complex (ABC) technique. Sections were first deparaffinized in xylene and rehydrated through a descending ethanol gradient. Endogenous peroxidase activity was blocked by incubating the sections in 0.3% hydrogen peroxide for 30 minutes at room temperature. Antigen retrieval was performed by heating the sections in 0.01 M sodium citrate buffer (pH 6.0) using a microwave oven for 10 minutes.

The sections were then incubated overnight at 4°C with the primary antibodies: anti-TNF-α antibody (Dako, Carpinteria, California, USA), anti-p53 mouse monoclonal antibody (DO-7, 1:50 dilution; Dako, Glostrup, Denmark), and rabbit polyclonal anti-COX-2 antibody (ab15191; Abcam, Massachusetts, USA).

After rinsing with phosphate-buffered saline (PBS), sections were incubated with a biotinylated secondary antibody for 30 minutes, followed by a 30-minute incubation with streptavidin–peroxidase complex. Immunoreactivity was visualized using diaminobenzidine (DAB) as the chromogen, and counterstaining was performed with Mayer’s hematoxylin. Negative controls were included by omitting the primary antibody.

Microscopic examination and digital imaging of stained sections were performed using a Leica ICC50 digital camera attached to a Leica DM500 light microscope (Leica Microsystems, Switzerland) at the Histology and Cell Biology Department, Faculty of Medicine, Kafrelsheikh University.

**Morphometric analysis**

Quantitative assessment of immunohistochemical staining was conducted on ten randomly selected, non-overlapping microscopic fields from each slide per group. The evaluation focused on calculating the mean area percentage of positively stained cells for TNF-α, p53, and COX-2 in DAB-labeled sections. ImageJ software (National Institutes of Health, Bethesda, MD, USA) was used for image analysis at 400× magnification, ensuring objective and reproducible measurements (Suvarna et al. 2018; Schneider et al. 2012).

**Quantitative reverse transcription polymerase chain reaction (qRT-PCR)**

**RNA extraction**

Total RNA was isolated from 30 mg of colonic tissue homogenized in 600 μL of RLT buffer (RNA Safeguard, Bioflux), supplemented with 10 μL β-mercaptoethanol per 1 mL. Homogenization was performed using a Qiagen TissueLyser set to 30 Hz for 2 minutes. RNA extraction was completed using the QIAamp RNeasy Mini Kit (Qiagen GmbH, Germany), following the manufacturer’s instructions. Subsequently, 70% ethanol (1:1 volume) was added to the lysate to facilitate RNA binding and purification.

**Primer design and PCR reaction**

Oligonucleotide primers were synthesized by Metabion (Germany), with specific forward and reverse sequences listed in (**supplementary Table S2**). Each 25 μL PCR reaction mixture contained 12.5 μL of 2× QuantiTect SYBR Green PCR Master Mix (Qiagen GmbH, Germany), 0.25 μL RevertAid Reverse Transcriptase (200 U/μL, Thermo Fisher Scientific), 0.5 μL of each primer (20 pmol), 8.25 μL nuclease-free water, and 3 μL RNA template.

Amplification was performed using the Stratagene Mx3005P real-time PCR system under the following conditions: initial denaturation at 94 °C for 15 seconds, followed by 40 cycles at 94 °C for 15 seconds, 60 °C for 30 seconds, and 72 °C for 30 seconds. Amplification curves and threshold cycle (Ct) values were analyzed using the system's software.

**Gene expression analysis**

Relative gene expression levels were normalized to β-actin as the housekeeping gene, and fold changes were calculated using the 2^−ΔΔCt method, as described by Yuan (2006). Expression levels in the control group were used as the baseline and set to one for comparison purposes across all groups (Bangaru et al. 2012; Livak & Schmittgen 2001).

**Measurement of TNF-α**

Colonic tissues were excised and rinsed thoroughly with sterile normal saline to eliminate any residual contents. A portion of each colon was then homogenized at a 10% weight/volume (w/v) ratio in a buffer containing 20 mM Tris-HCl and 1 mM EDTA (pH 7.4) using a glass homogenizer. The homogenate was subsequently sonicated and centrifuged at 3000 × g for 20 minutes at 4 °C. The resulting supernatant was collected and stored at −80 °C until analysis (Lima et al. 2019).

The concentration of tumor necrosis factor-alpha (TNF-α) in the colonic tissue was quantified using a commercially available ELISA kit specific for rats (Rat TNF-α ELISA Kit, Cat. No. ER1393; Fine Test, Wuhan Fine Biotech Co., China), following the manufacturer's protocol. This assay enabled accurate and sensitive detection of TNF-α as a marker of colonic inflammation.

**Assessment of oxidative stress biomarkers in colon homogenates**

To evaluate oxidative stress in colonic tissues, biomarkers including malondialdehyde (MDA) and total antioxidant capacity (TAC) were quantified in colon homogenates. Briefly, freshly excised colonic samples were washed with ice-cold saline, weighed, and homogenized in phosphate-buffered saline (PBS, pH 7.4) to obtain a 10% w/v homogenate. The homogenates were centrifuged at 3000 × g for 20 minutes at 4 °C, and the resulting supernatants were collected and stored at −80 °C until analysis (Erdemli et al. 2024).

Lipid peroxidation was assessed by measuring the concentration of malondialdehyde (MDA), a key marker of oxidative damage, using the OxiSelect™ TBARS Assay Kit (Cell Biolabs Inc., San Diego, USA), following the manufacturer’s instructions. Results were expressed as nanomoles of MDA per milligram of protein.

Total antioxidant capacity (TAC) was determined using the OxiSelect™ TAC Assay Kit (Cell Biolabs Inc., San Diego, USA), which quantifies the cumulative antioxidant activity against a defined oxidant challenge. Absorbance readings were obtained using a microplate reader, and values were expressed as µmol trolox equivalents per mg protein (Sarawi et al. 2023).

Protein concentrations in all samples were measured using the Bradford assay to ensure normalization of oxidative stress marker levels.

**Assessment of gene expression levels of *β*–actin, Nrf2, Keap1, NF-κB, and HO-1 mRNA**

Total RNA was extracted from approximately 30 mg of colonic tissue using the QIAamp RNeasy Mini Kit (Qiagen GmbH, Germany) in accordance with the manufacturer’s protocol. Samples were homogenized in 600 μL RLT lysis buffer (containing β-mercaptoethanol) using the Qiagen TissueLyser system at 30 Hz for 2 minutes. After homogenization, one volume of 70% ethanol was added to the lysate, and RNA purification was completed using spin column-based separation. The purity and concentration of RNA were assessed spectrophotometrically using a NanoDrop™ device (Thermo Fisher Scientific, USA) (Han et al. 2020)

Complementary DNA (cDNA) was synthesized from 1 μg of total RNA using RevertAid Reverse Transcriptase (Thermo Fisher Scientific, USA) in a 20 μL reaction volume according to the manufacturer's instructions. Quantitative real-time PCR (qRT-PCR) was performed using a Stratagene Mx3005P Real-Time PCR System (Agilent Technologies, USA) with a total volume of 25 μL per reaction. Each reaction mixture contained 12.5 μL of 2× QuantiTect SYBR Green PCR Master Mix (Qiagen), 0.5 μL of each gene-specific primer (20 pmol), 0.25 μL of RevertAid Reverse Transcriptase, 8.25 μL of nuclease-free water, and 3 μL of cDNA template.

Gene-specific primers for **Nrf2**, **Keap1**, **NF-κB**, **HO-1**, and the housekeeping gene ***β*–actin** were designed based on published sequences and synthesized by Metabion (Germany). Primer sequences are listed in **Table S2)**

Thermal cycling conditions included an initial denaturation at 94 °C for 15 seconds, followed by 40 amplification cycles of denaturation at 94 °C for 15 seconds, annealing at 60 °C for 30 seconds, and extension at 72 °C for 30 seconds. Melting curve analysis was performed to verify the specificity of amplification.

Relative gene expression levels were calculated using the 2^−ΔΔCt method, with β-actin serving as the internal reference gene. All values were normalized to the mean expression level of the control group (Rofaeil & Mohamed 2020; Zhao et al. 2021).

**Assessment of gene expression levels of Fendrr and Neat1 lncRNAs**

Total RNA was isolated from approximately 30 mg of colon tissue using the **QIAamp RNeasy Mini Kit** (Qiagen GmbH, Germany), following the manufacturer’s protocol. Tissues were homogenized in 600 μL of RLT lysis buffer supplemented with β-mercaptoethanol using a **Qiagen TissueLyser** at 30 Hz for 2 minutes. The homogenates were centrifuged, and the supernatant was processed through spin columns for RNA purification. RNA purity and concentration were assessed spectrophotometrically using a **NanoDrop™ 2000** (Thermo Fisher Scientific, USA), ensuring an A260/A280 ratio of 1.8–2.0 (Zhao et al. 2021).

Complementary DNA (cDNA) was synthesized using **RevertAid Reverse Transcriptase** (Thermo Fisher Scientific, USA) from 1 μg of total RNA in a 20 μL reaction volume, according to the manufacturer's instructions. Quantitative real-time PCR (qRT-PCR) was performed using the **Stratagene Mx3005P Real-Time PCR System** with **2× QuantiTect SYBR Green PCR Master Mix** (Qiagen, Germany) in a total volume of 25 μL per reaction.

Each qPCR reaction consisted of 12.5 μL SYBR Green master mix, 0.5 μL of each gene-specific primer (20 pmol; synthesized by Metabion, Germany), 0.25 μL of reverse transcriptase, 8.25 μL nuclease-free water, and 3 μL cDNA. The thermal profile included initial denaturation at 94 °C for 15 seconds, followed by 40 cycles of denaturation at 94 °C for 15 seconds, annealing at 60 °C for 30 seconds, and extension at 72 °C for 30 seconds. Specificity of amplification was confirmed through melt curve analysis.

Primers targeting **Fendrr** (FOXF1 adjacent non-coding developmental regulatory RNA) and **Neat1** (nuclear paraspeckle assembly transcript 1) were designed based on published sequences and validated in rat tissue [**Table S2**] (Lewesy 2006).

The relative expression of Fendrr and Neat1 was normalized to the housekeeping gene ***β*-actin**. Expression changes were calculated using the comparative Ct method (2^−ΔΔCt) (Rofaeil & Mohamed 2020). Control group values were normalized to 1.0 to allow comparison across experimental groups.

**Statistical analysis**

All data were expressed as mean ± standard deviation (SD). Differences among groups were analyzed using one-way analysis of variance (ANOVA), followed by Tukey’s post hoc test to determine intergroup significance. A *p-value* less than 0.05 was considered statistically significant. All statistical analyses were conducted using IBM SPSS Statistics software, version 23.0 (IBM Corp., Armonk, NY, USA) (Benzarti et al. 2012).

**Data availability**

All data generated or analyzed during this study are included in this published article and its supplementary information files. Additional details are available from the corresponding author upon reasonable request.

**Author contributions**

M.H.., M.El-A., A.K., G.A. Conceptualization, M.H., M.M.M., M.M.A., M.El-A., A.K., G.A. Methodology, M.H., M.M.M., M.M.A., M.El-A., A.K., G.A. Investigation, M.H., M.M.M., M.M.A., M.El-A., A.K., G.A. Formal analysis, M.H., M.M.M., M.M.A., M.El-A., A.K., G.A. Data curation, M.M.M., M.M.A., M.El-A., A.K., G.A. Writing - original draft., M.El-A., A.K., G.A. Supervision. Finally**,** M.El-A., A.K., M.M.M., M.M.A., G.A. Visualization, Writing - Review & Editing.

**Funding**

This study was self-funded.

**Conflict of interest**

The authors declare that they have no conflict of interest.

**Clinical trial number:**

Not applicable.

**Correspondence and requests for materials** should be addressed to G.A.

**References**

Abdl Aziz FT, Temraz A, Hassan MA (2024) Metabolites profiling by LC-ESI-MS/MS technique and in-vitro antioxidant activity of *Bauhinia madagascariensis* Desv. and *Bauhinia purpurea* L. aerial parts cultivated in Egypt: a comparative study. Azhar Int J Pharm Med Sci 4(1): 169-188. DOI: 10.21608/aijpms.2023.212409.1215.

Abdelgawad FAM, El-Hawary SS, Abd El-Kader EM (2024). Phytochemical elucidation and effect of *Maesa indica* (Roxb.) sweet on alleviation of potassium dichromate-induced pulmonary damage in rats. Plants 13(3):338. <https://doi.org/10.3390/plants13030338>.

Attard E (2013) A rapid microtiter plate folin-ciocalteu method for the assessment of polyphenols. Cent Eur J Bio 8 (1):48-53. DOI: 10.2478/s11535-012-0107-3.

Bancroft JD, Layton C (2010) The Hematoxylins and Eosin. In: Bancroft’s Theory and Practice of Histological Techniques. 7th ed., Churchill Livingstone Elsevier, Oxford, UK.

Bangaru ML, Park F, Hudmon A, McCallum JB, Hogan QH (2012) Quantification of gene expression after painful nerve injury: validation of optimal reference genes. J Mol Neurosci 46(3):497-504. DOI: 10.1007/s12031-011-9628-x. Epub 2011 Aug 24. PMID: 21863315; PMCID: PMC3273664.

Ben Nejma A, [Nguir](https://pubmed.ncbi.nlm.nih.gov/?term=Nguir+A&cauthor_id=25813159) A, [Jannet](https://pubmed.ncbi.nlm.nih.gov/?term=Ben+Jannet+H&cauthor_id=25813159) HB, [Hamza](https://pubmed.ncbi.nlm.nih.gov/?term=Hamza+MA&cauthor_id=25813159)  MA, [Daïch](https://pubmed.ncbi.nlm.nih.gov/?term=Da%C3%AFch+A&cauthor_id=25813159) A, [Othman](https://pubmed.ncbi.nlm.nih.gov/?term=Othman+M&cauthor_id=25813159) M, [Lawson](https://pubmed.ncbi.nlm.nih.gov/?term=Lawson+AM&cauthor_id=25813159) AM (2015) New septanoside and 20-hydroxyecdysone septanoside derivative from *Atriplex portulacoides* roots with preliminary biological activities. Bioorg Med Chem Lett 25 (8):1665-1670. DOI: [10.1016/j.bmcl.2015.03.028](https://doi.org/10.1016/j.bmcl.2015.03.028).

Benzarti M, Rejeb KB, Debez, A, Messedi D, Abdelly C (2012) Photosynthetic activity and leaf antioxidative responses of *Atriplex portulacoides* subjected to extreme salinity. Acta Physiol Plant 34(5):1679–1688. DOI:[10.1007/s11738-012-0963-5](https://doi.org/10.1007/s11738-012-0963-5).

Chaturvedula VSP, Prakash I (2012) Isolation of stigmasterol and *β*-sitosterol from the dichloromethane extract of *Rubus suavissimus* Int Curr Pharm J 1(9):239-242. DOI:[10.3329/icpj. v1i9.11613](https://doi.org/10.3329/icpj.v1i9.11613).

Collins D (2022) A HPLC-ESI-MS/MS study of hydroxybenzoic acids and related derivatives in commercial seaweed biostimulants and their plant growth bioactivity, Institute for sustainable industries and livable cities,Victoria University, Melbourne, Australia.

El Said M, Amer M (1965) Oil, fats, waxes and surfactants. Anglo-Egyptian Bookshop, Cairo.

El-Shial EM, Kabbash A, El-Aasr M, El-Feky OA, El-Sherbeni SA (2023) Elucidation of natural components of *Gardenia Thunbergia* Thunb. leaves: effect of methanol extract and Rutin on non-alcoholic fatty liver disease. Molecules 28(2):879. <https://doi.org/10.3390/molecules28020879>.

El-Zawahry HME, Shakour ZTA, El-Shafei AA, El-tantawy ME (2025) Larvicidal and cytotoxic activity of *Cordyline terminalis* Kunth and its metabolites profiling via UPLC-MS/MS. Azhar Int J Pharm Med Sci 5(1):141-151. <https://doi.org/10.21608/aijpms.2024.256016.1246>.

Erdemli Z., [Gul](https://www.sciencedirect.com/author/55807973900/mehmet-gul)  M., Gokturk N., Kayhan E, Demircigil N., Ozsoy E.N., Bag HG, Erdemli ME (2024) Ameliorative effects of thymoquinone on the caspase 3, kidney function, and oxidative stress tartrazine-induced nephrotoxicity, Toxicon 241(107660) . <https://doi.org/10.1016/j.toxicon.2024.107660>.

Fabre N, Isabelle R, de Hoffmann E, Quetin-Leclercq J (2001) Determination of flavone, flavonol, and flavanone aglycones by negative ion liquid chromatography electrospray ion trap mass spectrometry. J Am Soc Mass Spectrom 12(6):707–715. DOI:[10.1016/S1044-0305(01)00226-4](https://doi.org/10.1016/S1044-0305(01)00226-4).

Han H, Liu L, Chen M, Liu Y, Wang H, Chen L (2020) The optimal compound reference genes for qRT-PCR analysis in the developing rat long bones under physiological conditions and prenatal dexamethasone exposure model. Reprod Toxicol 98:242–251. <https://doi.org/10.1016/j.reprotox.2020.09.005>.

Jain PS, Bari SB (2010) Isolation of lupeol, stigmasterol and campesterol from petroleum ether extract of woody stem of *Wrightia tinctoria*. Asian J Plant Sci 9(3):163-167. DOI:[10.3923/ajps.2010.163.167](https://doi.org/10.3923/ajps.2010.163.167).

Kiranmai M, Kumar CM, Ibrahim M (2011) Comparison of total flavonoid content of *Azadirachta indica* root bark extracts prepared by different methods of extraction, RJPBCS 2(3):254-261.

Lewsey J (2006) Medical statistics: A guide to data analysis and critical appraisal. Ann R Coll Surg Engl 88(6):603. DOI: 10.1308/003588406X117098g. PMCID: PMC1963749.

Li H-Y, [Wang](https://pubmed.ncbi.nlm.nih.gov/?term=Wang+SM&cauthor_id=19142897) S-M, [Liu](https://pubmed.ncbi.nlm.nih.gov/?term=Liu+HM&cauthor_id=19142897) S-M, [Bu](https://pubmed.ncbi.nlm.nih.gov/?term=Bu+SS&cauthor_id=19142897) S-S, [Li](https://pubmed.ncbi.nlm.nih.gov/?term=Li+J&cauthor_id=19142897) J, [Han](https://pubmed.ncbi.nlm.nih.gov/?term=Han+D&cauthor_id=19142897) D, [Zhang](https://pubmed.ncbi.nlm.nih.gov/?term=Zhang+MZ&cauthor_id=19142897) M-z, [Wu](https://pubmed.ncbi.nlm.nih.gov/?term=Wu+GY&cauthor_id=19142897) G-Y (2009) Separation and identification of purine nucleosides in the urine of patients with cancer by reverse phase liquid chromatography/electrospray tandem mass spectrometry, J Mass Spectrom. 44(5):641–651. DOI: 10.1002/jms. 1539.

Lima TA, [Marton](https://www.researchgate.net/scientific-contributions/Ledyane-Taynara-Marton-2140337001?_tp=eyJjb250ZXh0Ijp7ImZpcnN0UGFnZSI6InB1YmxpY2F0aW9uIiwicGFnZSI6InB1YmxpY2F0aW9uIn19) LT, [Vaz de Marqui](https://www.researchgate.net/scientific-contributions/Samylla-Vaz-De-Marqui-2140313479?_tp=eyJjb250ZXh0Ijp7ImZpcnN0UGFnZSI6InB1YmxpY2F0aW9uIiwicGFnZSI6InB1YmxpY2F0aW9uIn19) S, [Fernando Cunha Neto](https://www.researchgate.net/scientific-contributions/Fernando-Cunha-Neto-2140325032?_tp=eyJjb250ZXh0Ijp7ImZpcnN0UGFnZSI6InB1YmxpY2F0aW9uIiwicGFnZSI6InB1YmxpY2F0aW9uIn19) FC (2019) The role of resveratrol in the inflammatory bowel diseases. Pharmacog Rev 13(26):36-44. DOI:[10.5530/phrev.2019.2.2](https://doi.org/10.5530/phrev.2019.2.2).

Livak KJ, Schmittgen TD (2001) Analysis of relative gene expression data using real-time quantitative PCR and the 2^−ΔΔCT method. Methods 25(4):402–408. <https://doi.org/10.1006/meth.2001.1262>.

Millar AD, [Rampton](https://pubmed.ncbi.nlm.nih.gov/?term=Rampton+DS&cauthor_id=8949646) DS, [Chander](https://pubmed.ncbi.nlm.nih.gov/?term=Chander+CL&cauthor_id=8949646) CL, [Claxson](https://pubmed.ncbi.nlm.nih.gov/?term=Claxson+AW&cauthor_id=8949646) AW, [Blades](https://pubmed.ncbi.nlm.nih.gov/?term=Blades+S&cauthor_id=8949646) S, [Coumbe](https://pubmed.ncbi.nlm.nih.gov/?term=Coumbe+A&cauthor_id=8949646) A, [Panetta](https://pubmed.ncbi.nlm.nih.gov/?term=Panetta+J&cauthor_id=8949646) J, [Morris](https://pubmed.ncbi.nlm.nih.gov/?term=Morris+CJ&cauthor_id=8949646) CJ, [Blake](https://pubmed.ncbi.nlm.nih.gov/?term=Blake+DR&cauthor_id=8949646) DR (1996) Evaluating the antioxidant potential of new treatments for inflammatory bowel disease using a rat model of colitis. Gut 39(3):407-415. DOI: 10.1136/gut.39.3.407.Suvarna K.S., Layton C., Bancroft J.D., Bancroft’s Theory and practice of histological techniques. 8th ed., Oxford, UK, Elsevier, 2018.

Mohammed HA, [Khan](https://pubmed.ncbi.nlm.nih.gov/?term=%22Khan%20RA%22%5bAuthor%5d) RA, [Abdel-Hafez](https://pubmed.ncbi.nlm.nih.gov/?term=%22Abdel-Hafez%20AA%22%5bAuthor%5d) AA, [Abdel-Aziz](https://pubmed.ncbi.nlm.nih.gov/?term=%22Abdel-Aziz%20M%22%5bAuthor%5d) M,  [Ahmed](https://pubmed.ncbi.nlm.nih.gov/?term=%22Ahmed%20E%22%5bAuthor%5d) E, [Enany](https://pubmed.ncbi.nlm.nih.gov/?term=%22Enany%20S%22%5bAuthor%5d) S, et al. (2021) Phytochemical profiling, in vitro and in silico anti-microbial and anti-cancer activity evaluations and Staph Gyrase B and h-TOP-II β receptor-docking studies of major constituents of *Zygophyllum coccineum* L. Aqueous-ethanolic extract and its subsequent fractions: An approach to validate traditional phytomedicinal knowledge. Molecules 26(3): 577. DOI: [10.3390/molecules26030577](https://doi.org/10.3390/molecules26030577).

Rofaeil RR, Mohamed AS (2020) Gastroprotective effect of sitagliptin in experimentally induced peptic ulcer in rats. MJMR. 31(2):253–256.

Ryhage R., Sydow E. von (1963) Mass spectrometry of terpenes, Acta. Chem. Scand. 17(7):2025-2035.

Sarawi WS, Alhusaini AM, Alghibiwi HK, Alsaab JS, Hasan IH (2023) Roles of Nrf2/HO-1 and ICAM-1 in the protective effect of nano-curcumin against copper-induced lung injury. Int J Mol Sci 24(18):13975. <https://doi.org/10.3390/ijms241813975>.

Schneider CA, Rasband WS, Eliceiri, KW (2012) NIH Image to ImageJ: 25 years of image analysis. Nature Methods 9(7):671–675. <https://doi.org/10.1038/nmeth.2089>.

Sri Harsha PSC, Abdul Wahab R, Cuparencu C, Dragsted LO, Brennan LA (2018) Metabolomics approach to the identification of urinary biomarkers of pea intake. Nutrients 10(12):1911. DOI: [10.3390/nu10121911](https://doi.org/10.3390/nu10121911).

Stahl E (1962) Dünnschicht-Chromatographie. In: Stahl E, Ed. Modern methods of plant analysis/Moderne methoden der pflanzenanalyse, 5th ed., Berlin, Germany: Springer.

Stanojević L, Zvezdanović J, Danilović B, Cvetković D, Stanojević J, Ilić D, [Milorad](https://scindeks.ceon.rs/Related.aspx?artaun=159616) C (2018) The antioxidative and antimicrobial activity of the aqueous earth smoke (*Fumaria officinalis* L.) extract, Adv. Technol. 7 (2):31-40. UDC 582.675.5:615.322:66.061.

Suvarna KS, Layton C, Bancroft JD (2018) Bancroft’s theory and practice of histological techniques. 8th ed., London, UK, Elsevier.

Taylora VF, Marchb RE, Longerichc HP, Stadeyb CJ (2005) A mass spectrometric study of glucose, sucrose, and fructose using an inductively coupled plasma and electrospray ionization. Int J Mass Spectrom 243(1):71–84. DOI:[10.1016/j.ijms.2005.01.001](https://doi.org/10.1016/j.ijms.2005.01.001).

Yuan JS, Reed A, Chen F, Stewart CNJr (2006) Statistical analysis of real-time PCR data. BMC Bioinformatics 7(85). DOI [10.1186/1471-2105-7-85](https://doi.org/10.1186/1471-2105-7-85).

Yuan Y, Hou W, Tang M, Luo H (2008) Separation of flavonoids from the leaves of *Oroxylum indicum* by HSCCC. Chromatographia. 68(11):885–892. DOI:[10.1365/s10337-008-0859-0](https://doi.org/10.1365/s10337-008-0859-0).

Zhao Q, Pang G, Yang L, Chen S, Xu R, Shao W (2021) Long noncoding RNAs regulate the inflammatory responses of macrophages. Cells 11(1):5. DOI: 10.3390/cells11010005. PMID: 35011565; PMCID: PMC8750547.

**Figure SI: The total ion chromatogram (TIC) of arial part of APME**

| **Table S1. Negative mode LC-MS-MS analysis of APME** | | | | | | | |
| --- | --- | --- | --- | --- | --- | --- | --- |
| **Peak**  **no.** | **R_t_ (min.)** | **Detected metabolites** | **Precursor**  ***m/z* [M-H]^-1^** | **Empirical**  **formula** | **Error**  **(ppm)** | **MS-MS fragmentation** | **Ref.** |
| ***Organic acids*** | | | | | | | |
| **1** | 1.11 | Malic acid | 133.01425 | C_4_H_5_O_5_ | -0.3 | 71.01553:180, 114.99906:514, 133.01573:402 | (Abdl Aziz et al. 2024) |
| **2** | 1.26 | 2-Isopropylmalic acid | 175.0612 | C_7_H_11_O_5_ | -0.9 | 85.06741:178,115.0397:399157.05688:178,  175.06306:1086 | (Sri Harsha et al. 2018) |
| **3** | 1.19 | D-3-Phenyllactic acid | 165.05573 | C_9_H_9_O_3_ | -5.9 | 75.01036:107,129.02437:71,147.03658:108,  165.03845:700 | (Abdl Aziz et al. 2024) |
| **4** | 2.91 | *p*-Hydroxybenzoic acid | 137.02441 | C_7_H_5_O_3_ | 0.6 | 93.03345, 137.02437:143 | (Collins 2022) |
| **5** | 4.46 | 2,5-dihydroxybenzoic acid | 153.01933 | C_7_H_5_O_4_ | 0.5 | 109.02848:156, 153.01823:393 | (Collins 2022) |
| ***Cinnamic acid derivatives*** | | | | | | | |
| **6** | 1.20 | Caffeic acid | 179.03499 | C_9_H_7_O_4_ | 0.2 | 89.02802:71,135.04303:107,179.05424:1584 | (Abdl Aziz et al. 2024) |
| 7 | 4.58 | Rosmarinic acid | 359.07724 | C_18_H_15_O_8_ | 6.2 | 161.02512:60, 359.08377:60 | (Stanojević et al. 2018) |
| ***Flavonoids and their glycosides*** | | | | | | | |
| **8** | 1.15 | Myricetin | 317.0303 | C_15_H_9_O_8_ | 2.8 | 112.98323:1659,180.97031:957248.94586:75, 317.05164:322 | (Abdl Aziz et al. 2024) |
| **9** | 1.40 | Baicalein-7-*O*-glucuronide (Baicalin) | 445.07764 | C_21_H_17_O_11_ | -0.7 | 102.95898:215,210.84923:72,445.07706:107 | (Abdl Aziz et al. 2024) |
| **10** | 5.14 | Kaempferol-3-*O*-*α*-L-rhamnoside (Afzelin) | 431.09836 | C_21_H_19_O_10_ | 0.4 | 179.0584:79,431.18436:393 | (Abdl Aziz et al. 2024) |
| **11** | 6.24 | Isorhamnetin-3-O-rutinoside (Narcissin) | 623.16174 | C_28_H_31_O_16_ | -1.9 | 307.07068:54, 623.11951:384 | (Abdl Aziz et al. 2024) |
| ***Continue Table S1*** | | | | | | | |
| **12** | 6.45 | Okanin-4'-O-glucosid | 449.10895 | C_21_H_21_O_11_ | 6 | 449.14368:215 |  |
| **13** | 6.81 | Kaempferol-7-neohesperidoside | 593.15118 | C_27_H_29_O_15_ | 2.8 | 593.15112:191 |  |
| **14** | 7.81 | Kaempferol-3-glucuronide | 461.07254 | C_21_H_17_O_12_ | 5 | 78.02939, 461.10397:308 | (Fabre et al.2001; Yuan et al. 2008) |
| **15** | 10.7 | 3,5, 7-Trihydroxy-4'-methoxyflavone  (Kaempferide) | 299.05612 | C_16_H_11_O_6_ | -1.9 | 284.0372:107, 299.05606:431 | (Abdl Aziz et al. 2024) |
| **16** | 13.22 | Procyanidin B2 | 577.13513 | C_30_H_25_O_12_ | 3.7 | 354.93893:71, 577.09814:71 |  |
| ***Carbohydrates and sugar derivatives*** | | | | | | | |
| **17** | 1.18 | L-(+)-Tartrate | 149.00916 | C_4_H_5_O_6_ | 1.7 | 71.01666:214,89.02392:605, 131.04077:480,  149.04547:1971 | (Abdl Aziz et al. 2024) |
| **18** | 1.18 | Gluconate | 195.05103 | C_6_H_11_O_7_ | 1.6 | 129.01151:143,129.01791:250,194.42003:146, 195.04868:938 | (Abdl Aziz et al. 2024) |
| **19** | 1.2 | Mucate | 209.0309 | C_6_H_9_O_8_ | -4 | 85.03104:366,89.02526:71,115.03224:72 129.01657:647,141.02219:107,159.033:254, 209.06667:2022 | (El-Zawahry et al. 2025) |
| **20** | 1.26 | Galactinol dihydrate | 378.10895 | C_12_H_25_O_13_ | 0.7 | 161.04321:71,179.06052:214,283.06073:143, 341.10687:447 | (Abdelgawad et al. 2024) |
| **21** | 1.43 | Sucrose | 341.10895 | C_12_H_21_O_11_ | 0.7 | 113.02077:107,119.03659:214,149.0406:71 161.04678:179,179.05673:401,341.01828:147, 341.10696:1293 | (Taylora et al. 2005) |
| **22** | 10.6 | Nystose Trihydrate | 665.2783 | C_24_H_41_O_21_ | -4.3 | 664.85736:89 |  |
| ***Purines and Pyrimidines*** | | | | | | | |
| **23** | 1.16 | Inosine | 267.07349 | C_10_H_11_N_4_O_5_ | -1.2 | 267.06076:107 |  |
| ***Continue Table S1*** | | | | | | | |
| **24** | 1.26 | Xanthosine | 283.06842 | C_10_H_11_N_4_O_6_ | 3.7 | 108.02817:74,151.02815:1267,239.08347:74 283.07022:684 | (Li et al. 2009) |
| **25** | 3.3 | 2'-Deoxyuridine-5'-monophosphat | 307.0423 | C_9_H_12_N_2_O_8_P | -0.2 | 307.08304:71 |  |
| **26** | 4.74 | Uridine 5'-monophosphate | 323.1175 | C_9_H_12_N_2_O_9_P | 0.5 | 323.11319:289 |  |
| **27** | 5.01 | UDP-xylose | 535.03717 | C_14_H_22_N_2_O_1_P_2_ | -0.5 | 535.14558:461 |  |
| **28** | 5.09 | 2’-Deoxyuridine-5’-triphosphatesodium salt | 466.96634 | C_9_H_15_N_2_O_14_P_3_ | 5 | 467.15594:214 |  |
| **29** | 5.5 | Uridine 5'-diphosphate | 403.1598 | C_9_H_14_N_2_O_12_P_2_ | 0.5 | 403.15765:79 |  |
| **30** | 5.7 | Thymidine-3’,5’cyclic monophosphate  sodium salt pyrimidine deoxynucleoside | 303.03876 | C_10_H_13_N_2_O_7_P | -0.4 | 303.08997:250 |  |
| **31** | 6.71 | Uridine5’-diphosphoglucuronic acid | 579.02704 | C_15_H_22_N_2_O_18_P_2_ | -2 | 579.13538:528 |  |
| ***Miscellaneous metabolites*** | | | | | | | |
| **32** | 1.18 | ˠ-Terpinene | 135.11792 | C_10_H_16_ | -1.1 | 135.02621:250 | (Ryhage & Sydow 1963) |
| **33** | 1.19 | L-5-Oxoproline | 128.03532 | C_5_H_7_NO_3_ | 0.9 | 128.03516:1859 |  |
| **34** | 1.49 | Ttrans-4-Hydroxy-L-proline | 130.05096 | C_5_H_9_NO_3_ | -0.6 | 130.08748:179 |  |
| **35** | 8.11 | L-*β*-homoisoleucine | 144.103 | C_7_H_15_NO_2_ | 1.2 | 144.04691:570 |  |

**Table S2: Sequences of primers used for RT-PCR**

| **Ref** | **Reverse** | **Forward** | **Accession No.** | **Gene** |
| --- | --- | --- | --- | --- |
| (Rofaeil & Mohamed 2020) | GCTCAGTAACAGTCCGCCTAGAA | TCCTCCTGAGCGCAAGTACTCT | NM_007393.5 | *β*–actin |
| * | TGAGTGTTAGGGATGCGCCC | CCTCCTGCACGTGAACTGGA | NR_126575 | FOXF1 adjacent non-coding developmental regulatory RNA (FENDRR) |
| * | TCTCCTTGCCAAGCTTCCTTC | TGGCTAGCTCAGGGCTTCAG | NR_028272.1 | Nuclear paraspeckle assembly transcript 1 (Neat 1) |
| * | CATGTCGAGGAAGACACTGGA | TTCCCTGAAGTGGAGCTAGGA | NM_199267.2 | NFĸB p65 |
| (Zhao et al. 2021) | CTACAAATGGGAATGTCTCTGC | CACATCCAGACAGACACCAGT | NM_031789 | Nuclear factor, erythroid derived 2, like 2 (Nrf2) |
| (Zhao et al. 2021) | TCGTCTCGATCTGGCTCATA | GGACGGCAACACTGATTC | NM_057152 | Kelch-like ECH-associated protein 1 (Keap 1) |
| (Zhao et al. 2021) | CTGTGAGGGACTCTGGTCTTTG | ACAGGGTGACAGAAGAGGCTAA | NM_012580 | Heme oxygenase (HO-1) |

*Désigne in the current study
